# Epigenetic and metabolic reprogramming of innate immune cells establishes immunological memory in the Schistosomiasis vector snail *Biomphalaria glabrata*

**DOI:** 10.1371/journal.ppat.1014471

**Published:** 2026-08-05

**Authors:** Rémi Pichon, Silvain Pinaud, Cristian Chaparro, Manon Fallet, Ricardo Lebron, Jean-François Allienne, Evgenia Turtoi, Christoph Grunau, Andrei Turtoi, David Duval, Richard Galinier, Céline Cosseau, Benjamin Gourbal

**Affiliations:** 1 IHPE, Univ Montpellier, CNRS, IFREMER, Université de Perpignan Via Domitia, Perpignan, France; 2 MIVEGEC (UM-CNRS 5290-IRD 224), IRD, Institute for Sustainable Development Agropolis Campus, Montpellier, France; 3 Man-Technology-Environment research centre (MTM), School of Science and Technology, Örebro University, Örebro, Sweden; 4 Department of Genetics, Faculty of Science, University of Granada, Campus de Fuentenueva s/n, Granada, Spain; 5 Laboratory of Bioinformatics, Centro de Investigación Biomédica, Granada, Spain; 6 Institut de Recherche en Cancérologie de Montpellier (IRCM), INSERM, Université de Montpellier, Montpellier, France; 7 Gunma University Initiative for Advanced Research (GIAR), Maebashi, Gunma, Japan; Texas Biomedical Research Institute, UNITED STATES OF AMERICA

## Abstract

Innate immune memory enables non-vertebrates to mount faster and more effective immune responses upon re-exposure to a previously encountered pathogen, yet its cellular and molecular bases remain poorly understood. The freshwater snail *Biomphalaria glabrata*, intermediate host of the human parasite *Schistosoma mansoni*, provides a powerful model to investigate this phenomenon. Here, we show that innate immune memory in *B. glabrata* is carried by hemocytes and relies on profound metabolic and epigenetic reprogramming initiated during primary infection. Using an integrative multi-omics approach combining transcriptomics, chromatin accessibility profiling, whole-genome bisulfite sequencing and targeted metabolomics, we reveal that the first parasite encounter induces a stable rewiring of hemocyte metabolism and chromatin landscape. This reprogramming primes hemocytes for a massive and rapid transcriptional response upon secondary challenge, characterized by an immune shift toward highly specific humoral effector pathways. Metabolic analyses demonstrate an early switch toward aerobic glycolysis, altered tricarboxylic acid cycle activity and amino acid metabolism, consistent with a Warburg-like metabolic state previously described in vertebrate trained immunity. Notably, metabolic and epigenetic remodeling occurs primarily during the primary infection and remains stable upon secondary exposure, suggesting that immune memory is encoded prior to pathogen re-encounter. Together, our results identify conserved metabolic and epigenetic mechanisms underlying innate immune memory in a non-vertebrate host and provide direct evidence that hemocyte-mediated innate immune memory in *B. glabrata* shares core features with trained immunity described in vertebrates.

## Author summary

The Fresh water snail *Biomphalaria glabrata* acts as the intermediate host of Schistosome parasites, a major human parasitic disease affecting millions of people worldwide. Understanding how the snail interacts with the parasite is key for finding new strategies to control schistosomiasis by targeting the parasite’s life cycle within its snail host. For such purpose, intense researches have been dedicated to the deciphering of the immunobiological interactions between both protagonists. Recently, studies showed that the snail *Biomphalaria glabrata* can develop a form of “immune priming” or “innate immune memory.” We demonstrate that after initial exposure to the parasite *Schistosoma mansoni*, these snails become strongly protected against subsequent infections. Herein, we found that this protection is linked to profound changes in snail’s immune cells. These include shifts in gene activity (transcriptomics), metabolism, and epigenetic regulation mechanisms that control how genes are turned on or off without altering DNA sequences. Together, these changes “reprogram” the immune system, enabling a faster and more efficient response upon re-exposure. These findings challenge the long-standing view that immunological memory is absent in non-vertebrates and highlight similarities with “trained immunity” observed in vertebrate organisms.

## Introduction

In the early 2000s the first demonstrations that non-vertebrates were able to develop immunological memory have shaken an old established paradigm that considered non-vertebrate immunity as relying solely on cellular non-sophisticated, and non-specific innate immune processes [1]. Indeed, over the past two decades, an accumulating number of studies have provided phenomenological evidence that non-vertebrates have the ability of inducing acquired-like immune response that can be specific and long-lasting, protecting them from subsequent exposures [2–7]. Invertebrate immune system could be “primed”, increasing the protection against secondary exposure to an already encountered pathogen [5,8–11]. These immunological memory responses have been named “immune priming” or “innate immune memory” (IIM) for non-vertebrates [2,5]. Even if some scepticism concerning IIM has been raised by some immunologists in the past (for the controversy see [3,12,13]) it is now clearly established that IIM is an overall general immune process of invertebrates, as it has been described in numerous taxa (arthropods, molluscs, nematodes, cnidarians) [2,5,14–17]. Such diversity of taxa is in line with the diversity of mechanisms underpinnings of IIM. Studies on IIM are indeed extremely heterogeneous in terms of invertebrate species studied; pathogen interaction used (bacteria, viruses and macro-parasites); elicitors used (lived or killed pathogens, active molecules or antigens); routes of inoculation (oral or injection); duration (from few days to months); and degrees of specificity (for review, see [5]) and mechanisms are thus dependant on such numerous criteria. Molecular data are increasingly studied in most cases for insect and mollusc models [15]. Consequently, the different mechanisms involved in IIM has to be deciphered for each model studied in order to gain a more realistic view of the immunobiological processes sustaining such phenomenon. It is thus impossible to come up with a universal description and more work is now needed to understand the nature and origin of these restricted, convergent, divergent or more or less conserved innovations, to get a comprehensive view of all these processes. Knowledge of the mechanisms underpinning IIM in non-vertebrates is currently just in its infancy.

Studies on the freshwater snail *Biomphalaria glabrata*, have provided some of the most fascinating examples for invertebrate IIM against the agent of human schistosomiasis [2,18]. Early studies conducted in the 1970s already reported that B. glabrata snails infected with trematodes like Echinostoma parasites, develop resistance to subsequent infections, a phenomenon referred to as “acquired resistance” [19]. It was only several years later that this phenomenon was demonstrated against Schistosoma mansoni [20]. Although the mechanisms underlying this resistance were unknown at the time, these observations strongly look like what is now described as innate immune priming. Indeed, snails that have previously been exposed to the parasite *Schistosoma mansoni*, showed increased resistance upon secondary exposure [11,18]. This IIM response is acquired in a time dependent manner, persists throughout the snail’s life (at least 140 days after primary infection) and is supported by a change from an immune cellular encapsulation response after primary infection toward a hemocyte-mediated humoral immune response after a secondary challenge [18]. Interestingly, the establishment of this immune memory coincides with parasite development, particularly the migration of daughter sporocysts to the digestive gland, suggesting a close link between parasite progression and long-term modulation of host immune responses. However, earlier studies have also shown that exposure to primary sporocyst (SpI) extracts is sufficient to induce a protective response [21], indicating that early parasite-derived signals can initiate immune priming. Using homologous and heterologous secondary challenges with *Schistosoma* parasite strains, it has been demonstrated a highly specific IIM response, involving polymorphic sets of immune recognition molecules and immune effector repertoires that are specifics to the pathogen encountered [21]. Finally, whole snail transcriptomic approach has demonstrated that this IIM response is supported by a so-far unreported process of immunological memory response called “immune shift” [18,22], involving a significant number of gene transcripts that are differentially expressed exclusively after the secondary challenge [18].

One hypothesis can potentially explain this transcriptional immune shift, namely an epigenetic reprogramming of the hemocytes, based on stable chromatin modifications or remodelling, some processes known to occur in non-vertebrates [23,24]. Moreover, recent studies have demonstrated that changes in cell metabolic pathways are important drivers of epigenetic reprogramming for innate immune cells in vertebrate trained immunity [25]. Accumulations of specific metabolites serve as signalling substrates or cofactors for chromatin-modifying enzymes [26]. Interestingly, similar metabolic changes have been demonstrated in *Biomphalaria* snails following primary pathogen infections [27–31], arguing for a pivotal role of cell metabolism in immunity and potentially for IIM acquisition.

Based on these previous results and considerations, we hypothesis that innate immune memory in *Biomphalaria glabrata* snails may be supported by metabolic and epigenetic reprogramming in hemocyte innate immune cells, resulting in transcriptional reprogramming and in the expression of an efficient immune response towards *S. mansoni* parasites. For answering this question, an integrative Omics approach was conducted to understand how hemocytes can record, store and activate an efficient immune response following a secondary encounter with a same pathogen. We investigated (i) hemocyte transcriptomic expression by RNAseq, (ii) DNA methylation by whole genome bisulfite sequencing (WGBS), (iii) assay for transposase-accessible chromatin sequencing (ATAC-Seq) to decipher the epigenetic reprogramming of hemocytes and (iv) metabolomics using mass spectrometry targeted approaches by liquid chromatography-high resolution mass spectrometry (LC-HRMS) to reveal specific metabolic pathway changes. Such molecular comparative approaches will be conducted for naive, primary infected and secondary challenged snails. The integration of all these data will be determinant to decipher the dynamic of hemocyte reprogramming in acquisition of IIM in *Biomphalaria glabrata* snails.

## Results

### Total hemocyte count and hemocyte sub-population numeration

**Fig 1:**
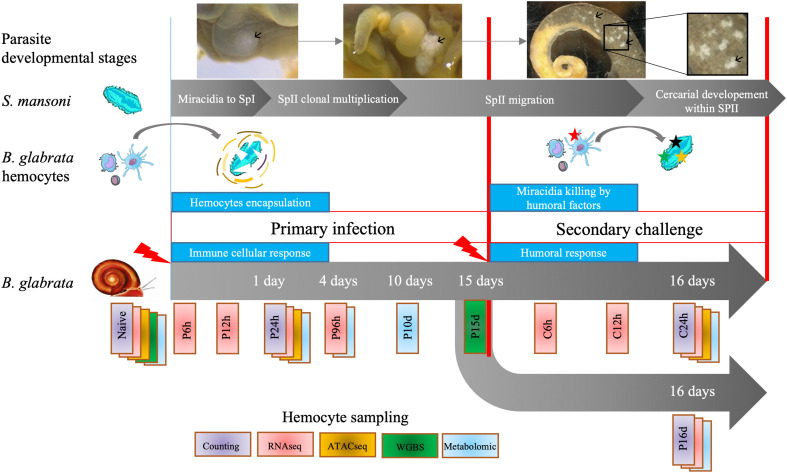
Experimental protocol. Infection kinetics used to characterize the innate immune memory response of the snail Biomphalaria glabrata from Brazil (BgBRE2) primary infected and secondary challenged with its sympatric Schistosoma mansoni parasite (SmBRE). The upper part of the Figure illustrates parasite development following primary infection and upon secondary challenge, together with associated hemocyte responses. After primary infection, miracidia penetrate the snail tegument; a majority are rapidly eliminated by hemocyte encapsulation, while the remaining parasites develop into primary sporocysts (SpI), which give rise to secondary sporocysts (SpII). Mature SpII subsequently migrate to the digestive gland to produce cercariae. Upon secondary challenge, invading miracidia are rapidly recognized and eliminated, and no reinfection occurs [11,18,20]. The lower part of the Figure shows hemocyte sampling time-points for counting, RNAseq, ATACseq, WGBS and metabolomic experiments were indicated along the kinetic of primary infection and secondary challenge using purple, pink, yellow, green, and blue rectangles respectively. Red flashes indicated the time point of primary and secondary infections using 10 miracidia of SmBRE.

**Fig 2:**
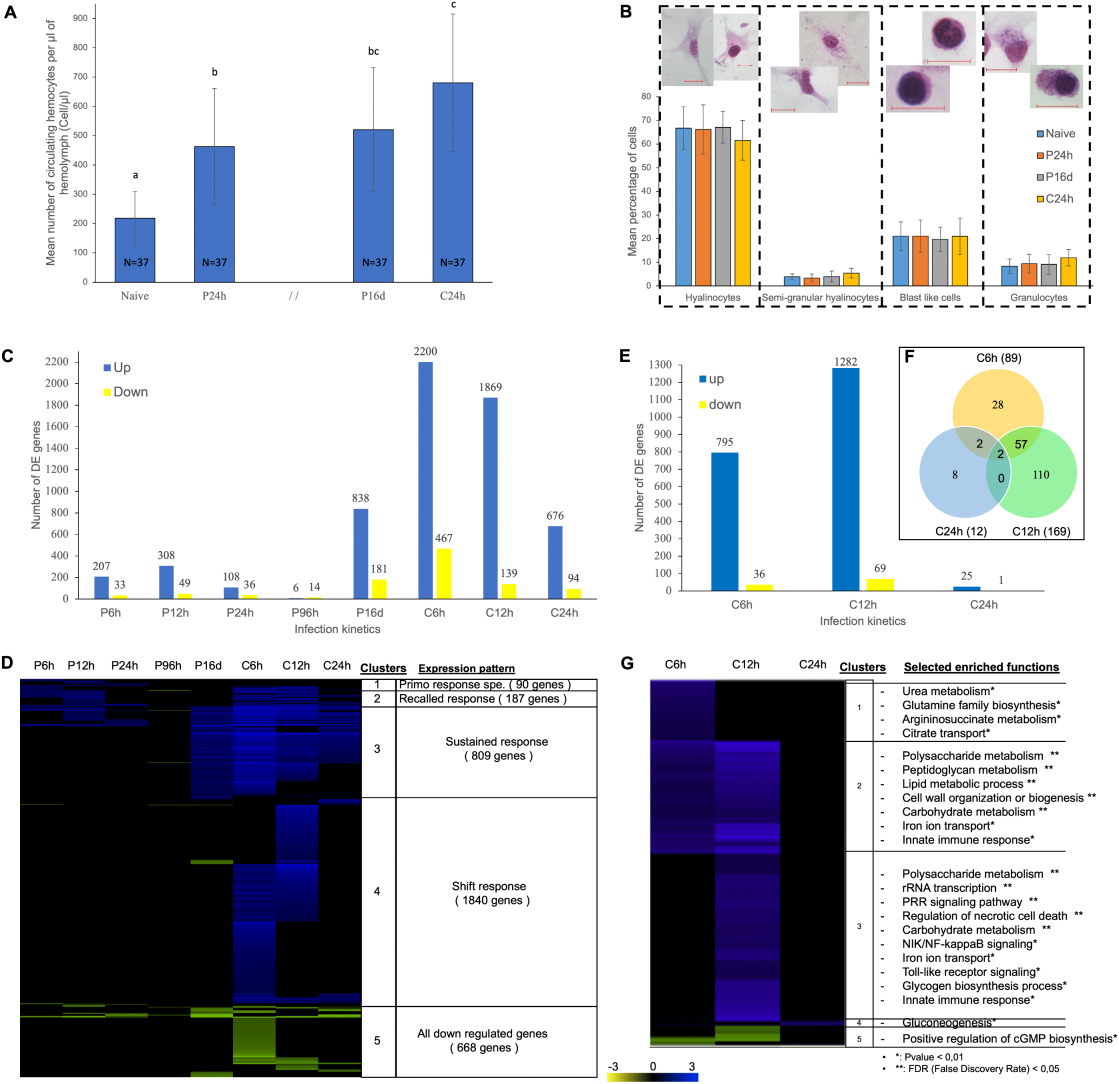
Changes in circulating hemocyte abundance and transcriptional reprogramming during IIM acquisition. **A.)** Mean number of circulating hemocytes per µl of hemolymph (Cell/µl) in BgBRE2 snails following primary infection and secondary challenge by SmBRE parasites. Error bars correspond to standard deviation. N: Number of snails used as replicates. Different letters (a, b, c) indicate statistical differences (P < 0.05) estimated by Dunn post-hoc test and FDR benjamini-Hochberg correction. **B.)** Representative morphological profiles of B. glabrata hemocyte populations stained with MCDh, red scale bars correspond to 10 µm. Associated graph show the mean percentage of each population according to infection kinetic time points (N = 10 per time point). **C.)** Histogram showing the number of differentially expressed genes (DEGs) in infected snails (primary infected and secondary challenged) compared with naïve snails during infection. Numbers above bars indicate the total number of significant DEGs. Blue and yellow represent up- and downregulated genes, respectively. **D.)** Heatmap of significant DEGs in infected snails compared with naïve snails during infection. Colours indicate log2 fold change (log2FC), with blue and yellow representing up- and downregulation, respectively. Genes are grouped into five clusters, depending on transcriptomic response patterns during the kinetics of infection. **E.)** Histogram showing the number of significant DEGs in secondary challenged snails compared with primary infected snails (P16d) over time. Numbers above bars indicate total DEGs. Blue and yellow represent up- and downregulated genes, respectively. **F.)** Venn diagram showing shared enriched functions among upregulated genes across the three conditions. **G.)** Heatmap of significant DEGs in secondary challenged snails compared with primary infected snails (P16d) during reinfection with SmBRE. Colours represent log2FC. Genes are grouped into five clusters, including four clusters of upregulated genes and one cluster of downregulated genes.

To avoid snail size effect on hemocyte counting, snails retained for all time points in the analysis (Fig 1) were selected for a size of 9 mm (S1 Table). Total hemocyte counts are shown in Fig 2A. The total number of hemocytes increased significantly following primary infection (noted “P”) and secondary challenge (noted “C”) compared to naïve snails (Kruskal-Wallis rank sum test: H = 54.02, df = 3, P-value = 1.11e-11; Dunn post-hoc Benjamini-Hochberg FDR method p-value < 0.05). As early as 24 hours after primary infection (P24h), the number of circulating hemocytes doubled and tended to increase until the end of the experiment, reaching 3.2 times more hemocytes at C24h than in naïve snails. However, the increase in circulating hemocytes between P24h vs P16d and P16d vs C24h was not significant.

Light microscopy approaches allowed us to characterize 4 distinct hemocyte populations called hyalinocytes, semi-granular hyalinocytes, blast-like cells, and granulocytes (Fig 2B). Hyalinocytes appeared as cells with large nucleus, long pseudopodia and high numbers of large cytoplasmic vacuoles without specific staining. Semi-granular hyalinocytes were similar to the hyalinocytes, with fewer light purple granulations in their cytoplasm (neutrophil granules). Blast-like cells are small and non-adherent cells with a round shape and a high nucleo/cytoplasmic ratio (often considered as pro-hemocytes). Finally, granulocytes are fairly large, spherical cells that produce small filopodia and whose cytoplasm always contains neutrophil granules (light purple) and basophil granules (dark blue) (Fig 2). There were no significant differences in counts of the 4 hemocyte populations across time points (Fig 2B). Thus, the increase of total hemocyte number (Fig 2A) was not associated with the proliferation of a specific hemocyte subtype but seems to be due to a proportional rise of all the hemocyte populations (Fig 2, S1 Table).

### Hemocytes transcriptomic expression profiles in the innate immune memory response

#### A limited subset of genes was regulated following the first 4 days after primary infection.

The results in Fig 2C indicate that the activation of the snail’s immune response against the parasite *S. mansoni* in hemocytes occurs over a period of 4 days. Interestingly, very few genes were differentially regulated in this early phase from P6h to P96h after the parasite infection (Fig 2C). Briefly, 207, 308, 108 and 6 genes were up-regulated at P6h, P12h, P24h and P96h respectively, compared to the naive control. Fewer than 50 genes were downregulated for the same time points. At 96 hours, the hemocyte response returns to baseline levels, which is interpreted as the end of the primary immune response against the SmBRE parasite.

Concerning gene functions and gene ontology (GO), most of the up-regulated genes during the first 96h of infection belonged to Pattern Recognition Receptor (PRR; i.e., c-type lectin), tumor necrosis factor (TNF) and antimicrobial proteins families. GO functions are mainly involved in cell movement and in different metabolic pathways, such as the urea cycle, lipid metabolism, carbohydrates metabolism, or TCA cycle with the enrichment of tricarboxylic acid transporters (S3 and S4 Tables). Functions of down-regulated genes are involved in hydrolase activity, calcium-ion transport or metalloprotease functions, and interestingly two down-regulated genes at P12h and P96h are cytochrome b and NADH dehydrogenase subunit, genes involved in the mitochondrial electron transport chain and cell energy metabolism through the oxidative phosphorylation (OxPhos) pathway (S3 Table). GO enrichment analysis could not be performed due to the insufficient number of differentially expressed genes (DEGs) between P6h and P96h that did not lead to statistically significant enriched functions in the GO term analysis.

#### A sustained immune response remains activated two weeks after primary infection.

After the return to a baseline state of the immune response at 96h, 1019 genes were detected as differentially regulated 2 weeks after infection (see P16d in Fig 2C and 2D) and most of these genes were maintained at a high level of expression after the secondary challenge (809 up-regulated genes and 81 down-regulated genes) (see cluster 3, Fig 2D). These genes, which are expressed during primary infection and whose expression remains at the same level after the secondary challenge, are considered to be part of the sustained immune response (Fig 2D).

GO terms associated with these genes were related to the following functions: cell and leukocyte differentiation, migration and proliferation; cell adhesion and communication; interleukin production and response; membrane signaling receptors; cell metabolism (TCA cycle, Pentose Phosphate Pathway (PPP)); production of Reactive Oxygen Species (ROS); and enzymatic/hydrolytic activities. GOs of down-regulated genes are mainly associated with carbohydrate and polysaccharide metabolism and catabolism of cell wall (S3 and S4 Tables).

#### Secondary challenge triggers a massive transcriptional reprogramming of hemocytes towards immune humoral functions and cellular energy metabolism.

A huge transcriptional response is observed after the secondary challenge (C6h to C24h, Fig 2C and 2D), representing 2285 DEGs (see Fig 2C and clusters 2, 3 and 4 in Fig 2D). Within those DEGs if we disregard genes involved in the sustained response described above (cluster 3, Fig 2D; 28.5% of the secondary challenge DEGs), the other DEGs were classified into 2 patterns of regulation yet described in previous studies [2,5,18,22,32]; i) DEGs displaying a recalled-response profile (cluster 2, Fig 2D; 6.6% of DEGs; genes regulated between P6h to P24h and similarly between C6h to C24h) and ii) DEGs displaying a shift-response profile (cluster 4, Fig 2D; 64.9% of the DEGs; genes that were regulated solely after the secondary challenge).

The recalled-response included up-regulated genes with functions related to lipid metabolism, organic substance catabolism, amino sugar metabolism or TCA cycle and down-regulated genes with functions related to the oxidative phosphorylation pathway (OxPhos) (S3 Table). In particular, we noticed that the genes encoding the NADH dehydrogenase subunit and cytochrome b (OxPhos pathway) were downregulated after primary infection by 2-fold and 2.8-fold respectively, and this downregulation was even stronger (4.3-fold and 7.0-fold) after the secondary challenge (S3 Table). This stronger response following the secondary challenge is observed for most of the DEGs associated with the recalled pattern for both up-regulated or down-regulated genes (Fig 2, S3 Table).

Concerning the shift-response to subtract the DEGs associated with the sustained response and concentrate exclusively into the characterization of DEGs solely regulated following the secondary challenge, we used the P16d as control to compared the level of genes expression from all the secondary challenged conditions (C6h, C12h and C24h) (Fig 2E, 2F and 2G). A total of 2208 genes exhibited differential expression in the secondary challenge compared to P16d sample (Fig 2E and 2G). The majority of DEGs were up-regulated (95%), while a small proportion was down-regulated (5%) (Fig 2E, 2F and 2G). Notably, a substantial change in the transcriptional profile was observed at C6h and C12h (831 and 1351 DEGs, respectively) (clusters 1, 2, and 3 of Fig 2G). This response was markedly attenuated at C24h with only 26 DEGs (cluster 4 Fig 2G).

The DEGs associated with the shift-response, are involved in immune functions related to tumor necrosis factor (TNF, BGLB023533, BGLB000225, BGLB012733, BGLB034099), toll-like receptor (TLR3: BGLB035706, TLR4: BGLB035763, BGLB036594, TLR8: BGLB025157, TLR13: BGLB038275), Thioester containing-protein family (BgTEP2: BGLB030043, BgC3-1: BGLB025256, BGLB021062, BGLB000085, BgC1q-like: BGLB037104, BgA2M: BGLB016521), FREPs (Fibrinogen-related proteins; BGLB000204, BGLB004530, BGLB007591, BGLB016746, BGLB020380), or biomphalysin (beta pore-forming toxin family members (Biomphalysin2: BGLB000033, biomphalysin13: BGLB000122; biomphalysin14: BGLB000086; biomphalysin15: BGLB000076)) (S3 Table). Enriched GO functions at C6h were related to the regulation of transcription, immune responses, self/non self-recognition (PRRs expression), cell movement or cytoskeleton remodeling, and metabolism-related functions (urea cycle, lipid metabolism and catabolism, TCA cycle and carbohydrate metabolism and glucose metabolism) (S4 Table). We anticipate that glycolysis is up-regulated since we observed that the hexokinase 2 (BGLB010911) encoding gene is up-regulated by 2.4-fold (S3 Table). A large part of the enriched functions observed at C6h remained enriched at C12h (57 out of 89 functions, Fig 2F). However, C12h was further enriched with other functions (GO cellular component) involved in vacuole, lytic vacuole, lytic enzyme or cytoskeleton remodeling (S4 Table). At C24h, the secondary challenge response seems to be over; only a few functions involved in immunity, cell differentiation and regulation of metabolism of different sugars remained enriched. Finally, we observed that the secondary challenge specific down-regulated genes at C12h and C24h, regroup mainly genes involved in the OxPhos pathway in the mitochondria (see cytochrome genes BGLB019393, BGLB019394, BGLB012929, BGLB018934; NADH dehydrogenase BGLB034537; and mitochondrial transporter BGLB010149, BGLB000601, BGLB021090 in S3 Table) and (see in S4 Table the GO functions highlighted in green in page “GO_functions_chall_down”).

**Fig 3:**
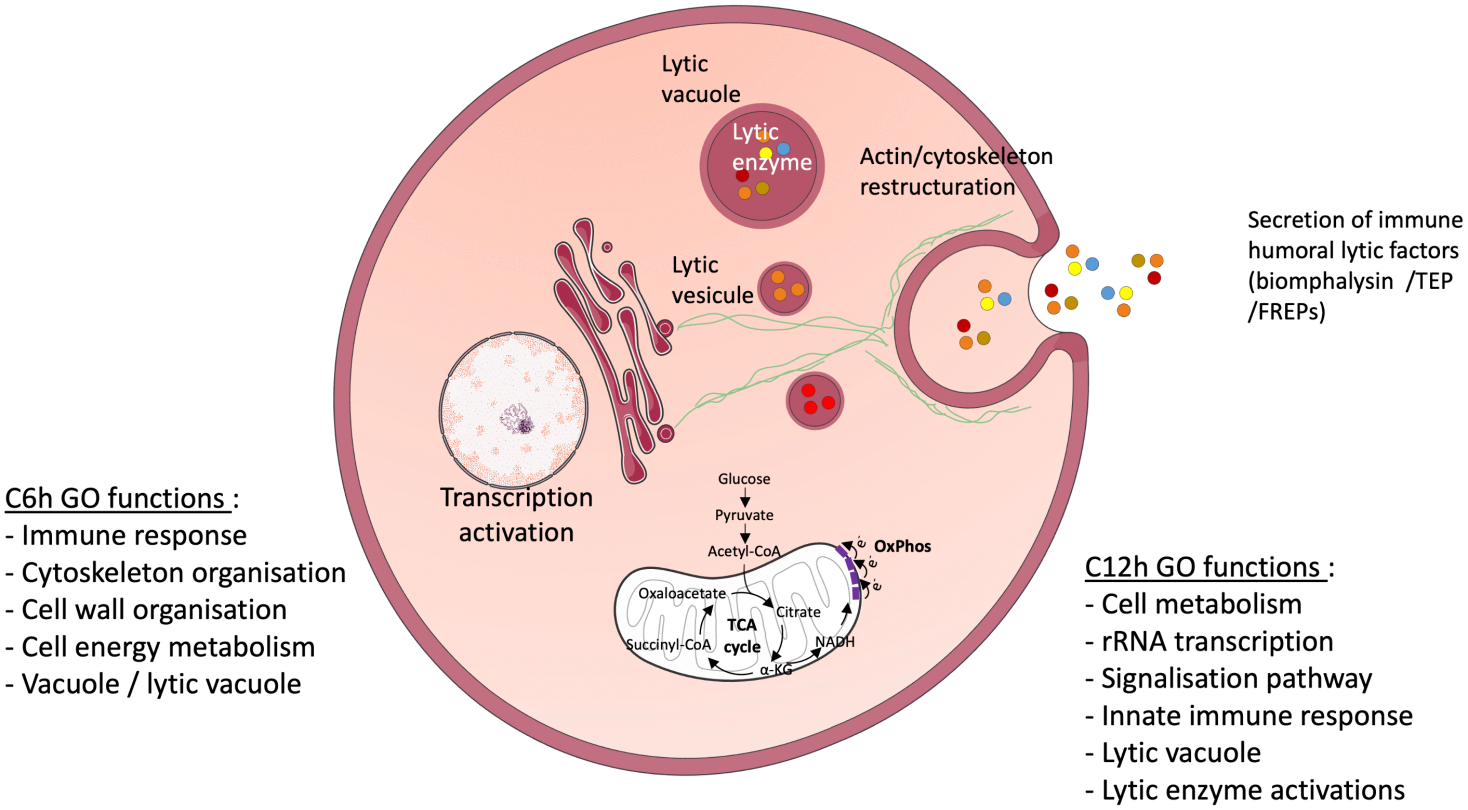
Schematic representation of hemocyte activation and response to secondary challenge infection.

To characterize transcriptional dynamics following secondary challenge, DEGs were classified into three categories based on their temporal expression profiles: recalled, sustained, and shift responses. Recalled genes show transient expression during primary infection and re-induction upon secondary challenge. Sustained genes are induced during primary infection and maintained at similar levels after the secondary challenge. Shift genes represent transcripts only expressed during secondary responses, reflecting a reprogramming of hemocyte functions. Following secondary challenge with *S. mansoni*, hemocytes of *B. glabrata* exhibited all three response types. The shift response was the most predominant, emerging as early as 6 h post-secondary challenge, peaking at 12 h, and declining by 24 h (Fig 2G). Functional annotation of these DEGs revealed significant changes between C6h and C12h, consistent with a cellular energy metabolism reorganization and a transition toward humoral immune response (Fig 3).

### A Whole Genome Bisulfite Sequencing (WGBS) to characterize the genomic methylation status of hemocytes following primary infection

**Fig 4:**
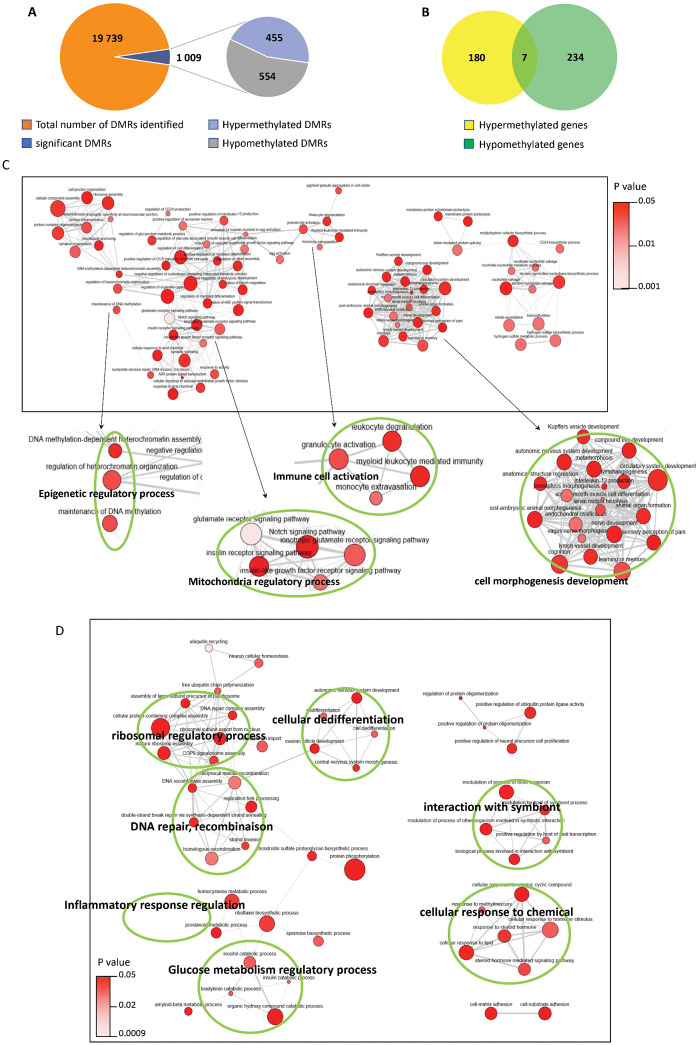
Differentially Methylated Genes in IIM and REVIGO Interactive graph view of hemocyte WGBS gene ontology functions. **A.)** Number of differentially methylated regions (DMRs) in hemocytes, indicating the numbers of hypomethylated DMRs and hypermethylated DMRs; **B.)** Venn diagram showing the numbers of hemocyte differentially methylated genes (DMGs) found as hypomethylated or hypermethylated in DMRs. **C.)** hypomethylated genes for GO corresponding to BP: Biological Process. **D.)** hypermethylated genes for GO corresponding to BP: Biological Process. Bubble color by a gradient of red to pink indicates the p-value according to the legend in upper right-hand corner (scale bar from 0.05 to 0.001); bubble size indicates the frequency of the GO term in the underlying GOA database (bubbles of more general terms are larger). Highly similar GO terms are linked by edges in the graph, where the line width indicates the degree of similarity. The placement of the nodes is determined by a ‘force-directed’ layout algorithm that aims to keep the more similar nodes closer together.

Due to the high number of snails required for Whole Genome Bisulfite sequencing (WGBS) (Table 1), this analysis was performed exclusively by comparing hemocyte DNA methylation profiles of 15 days primary infected snails (P15d) to naive snails. Hemocyte DNA methylation is predominantly in a CpG region and displayed a mosaic pattern, characterized by alternating methylated and unmethylated genomic regions, as previously described in whole snails [33]. The global level of hemocyte cytosine methylation was 1.17% and 1.14% (compared to all CpG) in naive and primary infected snails respectively, no significant difference was observed between the two conditions showing that the infection did not impact the level of cytosine methylation at the whole genome scale in hemocytes. This suggested that if certain regions of the genome are demethylated then other regions will be methylated in parallel. This led to a global balance of methylation across the entire hemocyte genome. To gain deeper insights into the impact of the infection by *S. mansoni* to identify Differentially Methylated Regions (DMRs) of hemocytes between primary infected and naive snails (Fig 4) [34]. The differential methylation analysis led to the detection of 1009 DMRs, among which 554 were hypomethylated and 455 regions were hypermethylated (S5 Table) (Fig 4A). This clearly showed that infection has an impact on the local pattern of cytosine methylation in hemocytes (Fig 4A). Next, we found that 420 DMRs intersected with gene positions, defining these regions as Differentially Methylated Genes (DMGs). Among those 421 DMGs, 180 genes were hypermethylated (HyperMGs), 234 genes were hypomethylated (HypoMGs) and 7 were impacted by both hyper- and hypo-methylations (Fig 4B). To go further, we investigated GO functions which were overrepresented among those HyperMGs and HypoMGs (Fig 4C and 4D, S6 Table). According to this analysis, the functions mostly impacted by DNA methylation changes were related to Immune cell activation and cell morphogenesis/ development, mitochondria regulatory process and epigenetic regulation for hypomethylated genes (Fig 4C, S6 Table). For hypermethylated genes, enriched GO functions were related to different cellular processes (dedifferentiation, response to chemical, DNA repair, recombination, ribosomal regulatory process), then more global metabolic processes were also identified belonging to interaction with symbiont, regulatory processes of glucose metabolism and regulation of the inflammatory response (Fig 4D, S6 Table).

**Table 1:** Overview of biological samples used for each experimental approach.

Experiment	Naive snails	Primary infected snails	Secondary challenged snails
**Total hemocyte count**	individual (N=37)	individual (N=37 per condition)	individual (N=37)
**Population hemocyte count**	individual (N=10)	individual (N=10 per condition)	individual (N=10)
**RNAseq**	pool of 12 (N=3)	pool of 12 (N=3 per condition)	pool of 12 (N=3 per condition)
**ATACseq**	pool of 10 (N=2)	pool of 10 (N=2)	pool of 10 (N=2)
**WGBS**	pool of 150 (N=3)	pool of 150 (N=3)	ND
**Metabolomic**	pool of 20 (N=5)	pool of 20 (N=5 per condition)	pool of 20 (N=5)

N: number of replicates: correspond to the number of biological replicates done for each experiment; conditions:correspond to the time points analysed in the kinetic of primary infection and secondary challenge.

It is interesting to note that we did not observe any canonical association between expression levels and methylation changes when analyzing correlations between DMGs and DEGs profiles (S11 Table). Indeed, solely 15.5% of hypermethylated genes and 10.4% of hypomethylated genes were also differentially regulated and among these genes we have both up- and down-regulation (S11 Table). The role of DNA methylation must therefore be sought elsewhere than at the level of transcriptomic regulation in *B. glabrata* snails.

### An ATAC-seq approach to investigate the hemocyte chromatin accessibility

**Fig 5:**
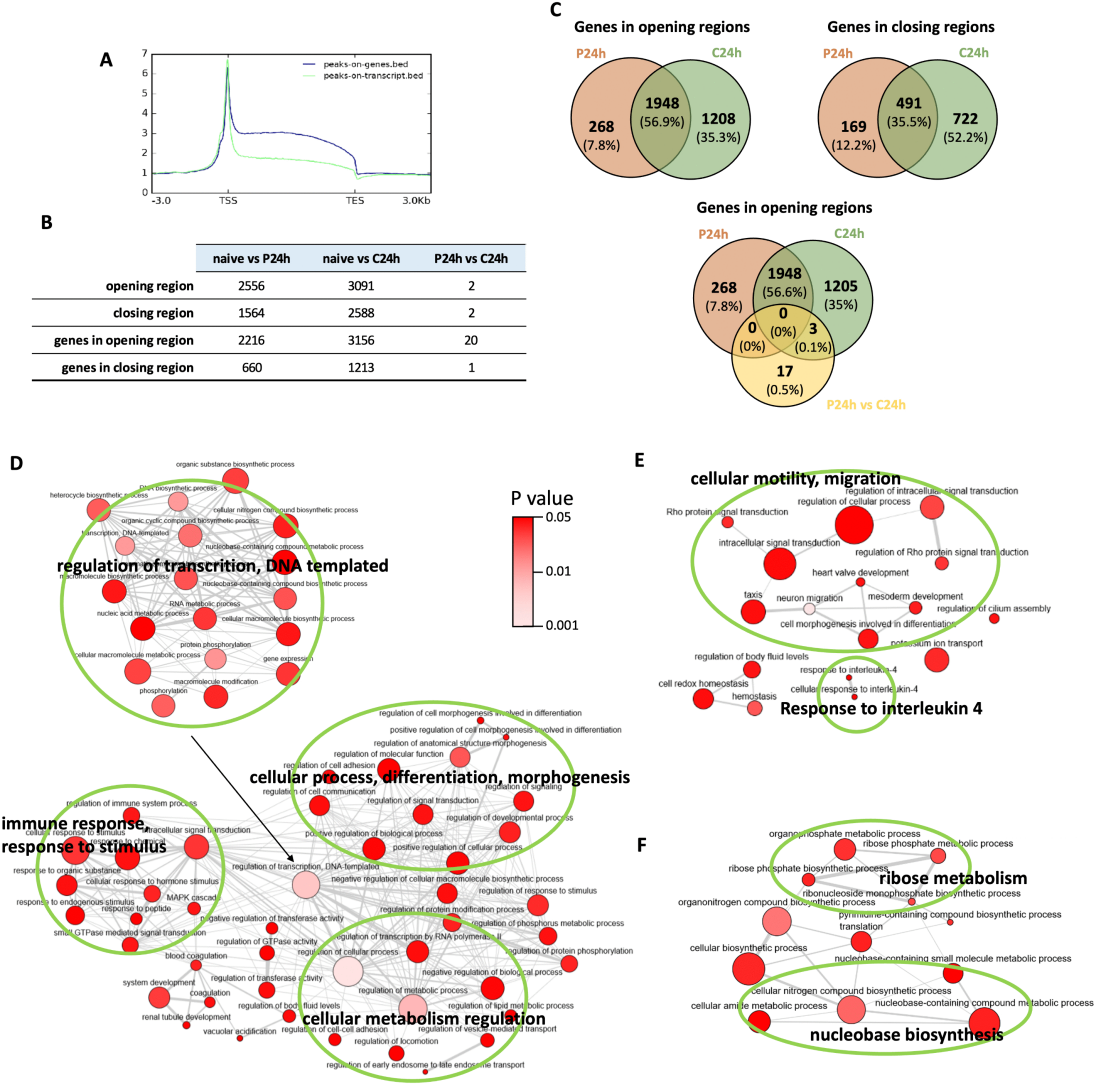
ATACseq data of hemocyte chromatin accessibility changes in IIM and REVIGO Interactive graph view of hemocyte ATACseq data for Biological Process GO enrichment. **A.)** Line plots showing the average chromatin accessibility levels around the TSS/TES ± 3 kb region in hemocytes. The blue line and green line indicate gene and transcript data, respectively; **B.)** summarizing changes in chromatin accessibility comparing Naive, P24h and C24h samples, indicating the total number of opening and closing genomics regions and the number of genes in these genomic regions; **C.)** Venn diagram showing the numbers of genes found in opening or closing regions shared between the different samples. **D.)** Genes in opening chromatin regions comparing naïve vs P24h/C24h samples, **E.)** Genes in closing chromatin regions comparing naïve vs P24h/C24h samples; **F.)** genes in opening chromatin regions comparing P24h vs C24h samples. Bubble color by a gradient of red to pink indicates the p-value according to the legend in upper right-hand corner (scale bar from 0.05 to 0.001); bubble size indicates the frequency of the GO term in the underlying GOA database (bubbles of more general terms are larger). Highly similar GO terms are linked by edges in the graph, where the line width indicates the degree of similarity. The placement of the nodes is determined by a ‘force-directed’ layout algorithm that aims to keep the more similar nodes closer together.

ATAC-seq allows us to follow changes in chromatin accessibility following primary infection and secondary challenge in hemocytes (Fig 5 and S7 Table). Comparing two experimental samples, the chromatin accessibility increases when a higher number of reads maps to the genome for a specific genomic region (i.e., opening genomic regions). Reciprocally, the chromatin accessibility decreases when the number of mapped reads for a genomic region decreases (i.e., closing chromatin regions) (Fig 5). This increase in mapped reads for a specific genomic region is related to an increase in the proportion of cells in the hemocyte population (cell population frequency) that displayed similar modifications of chromatin accessibility in corresponding genomic regions.

Our data shows that read peaks were transcript specific and were not distributed randomly along the transcript but were mainly located on the transcription start sites (TSS) (Fig 5A). This indicates clearly that the changes in chromatin structure and accessibility in hemocytes following infection were not random but finely regulated. This observation is strengthened by the fact that 94.3% of the genomic regions showing increased chromatin accessibility were associated with a predicted gene within these regions for P24h and C24h samples (Fig 5B). The situation is much less evident for closing genomic regions, indeed, decrease of chromatin accessibility seems more erratic as less than half of the closing regions (44.6%) are associated with a predicted gene (Fig 5B). We observed 2556 opening regions at P24h and 3091 opening regions at C24h, both compared to naive snail’s hemocytes. Those regions were associated with 2216 and 3156 genes respectively (Fig 5B). Comparing the same samples, 1564 and 2588 closing regions were identified that are associated with 660 or 1213 genes for P24h and C24h respectively (Fig 5B). Thus, we can see that chromatin accessibility changes drastically as early as P24h (Fig 5B). Chromatin accessibility continue to evolve after P24h, which explains why it is possible to detect some additional regions of chromatin that open or close when comparing C24h to the naïve sample, but the differences between peak values are not large enough to be significant when comparing the C24h and P24h samples (2 opening and 2 closing regions were significant (Fig 5B)). Changes in chromatin structure appeared to be mainly due to the primary infection.

Most of the genes that are associated with opening and closing regions were shared between P24h and C24h when compared to naïve sample (56.9% and 35.5% respectively) (Fig 5C). Only 17 genes (0.5%) were specifically associated with the secondary challenge (Fig 5C). Moreover, all the genes of P24h and C24h in opening and closing regions fall within the same GO functions and gave similar results following GO enrichment analysis (Fig 5D and 5E; S8 Table). GO functions over-represented among genes identified in opening chromatin regions for P24h and C24h were related to immune response/ response to stimulus, cellular differentiation and morphogenesis, cellular metabolism regulation and the regulation of transcription (Fig 5D; S8 Table). All these enriched functions fit perfectly with the phenotypic changes (i.e., a shift from a cellular response by hemocytes to a humoral response) that were expected to occur in hemocytes following primary infection and IIM acquisition. GO functions over-represented among genes identified in closing chromatin regions for P24h, C24h were related to cellular motility and migration and response to interleukin 4 (Fig 5E, S8 Table). The fact that motility and migration are associated with closing regions is not surprising if we consider that hemocytes are shifting from a cellular immune response toward a humoral immune response in the acquisition of IIM. Finally, the 20 genes that were associated with opening chromatin regions (Fig 5B) in C24h sample were mainly associated with ribose metabolism and nucleobase synthesis (Fig 5F). These enriched functions can be associated with the huge transcriptional activity observed in hemocytes after the secondary challenge.

Then, we investigated whether there was a canonical association between DEGs and their presence in opening or closing chromatin regions. Within 2216 genes in opening chromatin regions after the primary infection (Fig 5B), 13.3% (296 genes) were differentially regulated following the primary infection and 27.6% following the secondary challenge (S12 Table). Interestingly, 35.6% of the genes that fall into the sustained-response and 21.6% of the genes assuming a shift-response were associated to ATAC-seq opening regions (S3, S7 and S12 Tables), indicating that changes in chromatin accessibility seems to better supports the sustained-response in Innate Immune Memory. Concerning closing chromatin regions, no canonical association with DEGs could be observed. Solely 4.5% and 12.7% of the 660 genes associated to primary infection closing-chromatin-regions were differentially regulated following primary infection or secondary challenge respectively (S12 Table). As stated above, closing chromatin regions seem to not be tightly regulated, and might be the result of a mechanistic constraint that requires certain chromatin regions to be closed in order to specifically opened others, or regions corresponding to other genomic structures, like transposable elements, repeat regions. Answering this question will deserve further investigations.

### A targeted metabolomics approach to validate hemocyte metabolic reprogramming

**Fig 6:**
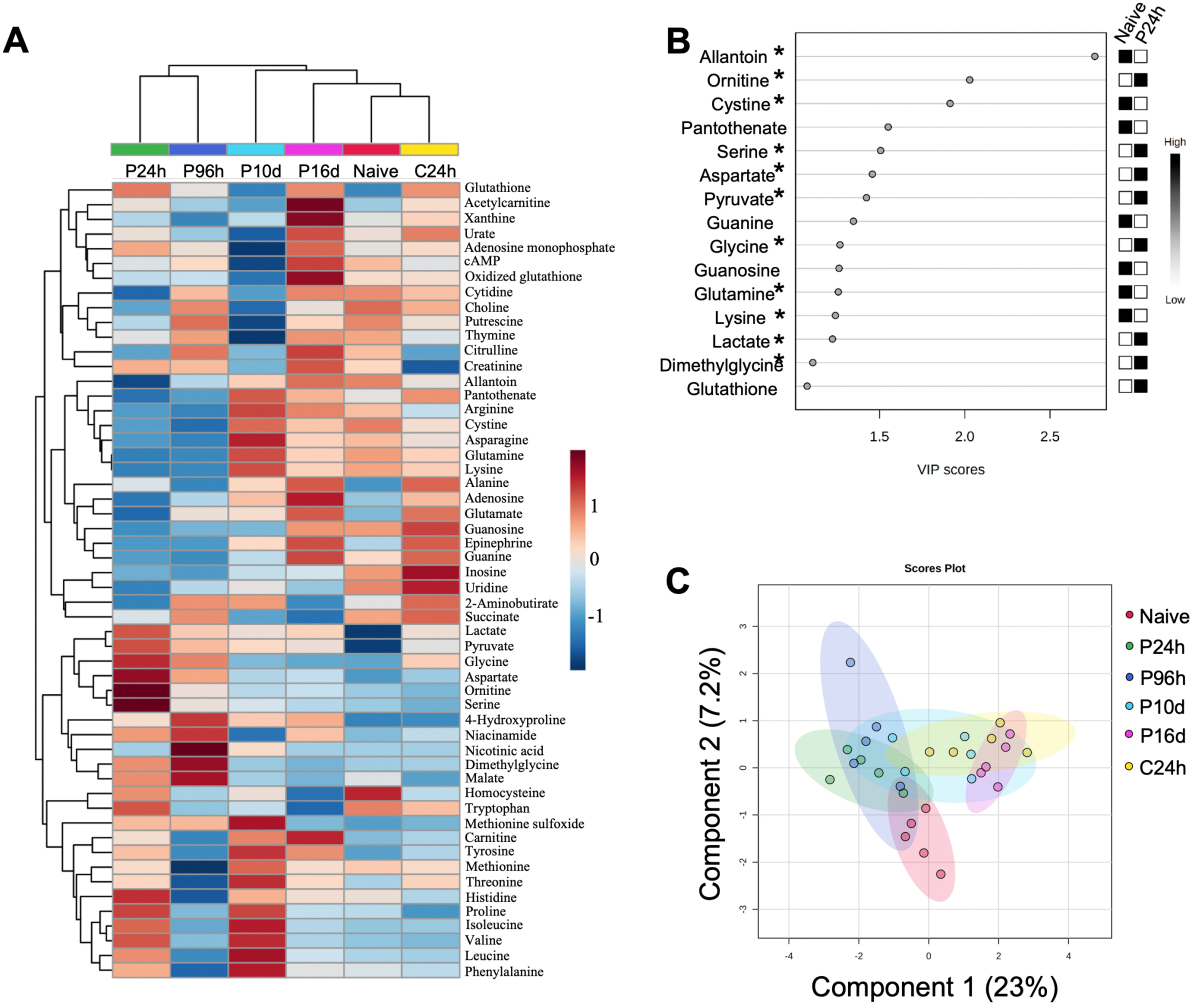
Targeted metabolomics analyses of hemocyte. **A.)** Heat-map representing in a blue and red gradient, the average quantity of each metabolite for each time points of the infection kinetic clustered by Euclidean distance. **B.)** Comparison of naive vs P24h samples, top 15 metabolites with the best VIP score (>1) (Variable Importance in the Projection) based on the PLS-DA model. Heat-map with white and black boxes represent high and low abundance ratio for the corresponding metabolite in the respective sample. **C.)** PLS-DA score clustering plot for the comparison of the 54 metabolites from the different kinetic times, grouping 5 replicates per condition (colored dots). Colored circles represent 95% confidence intervals. * Indicate VIP with significant difference (FDR < 0.05) comparing naïve to P24h.

**Fig 7:**
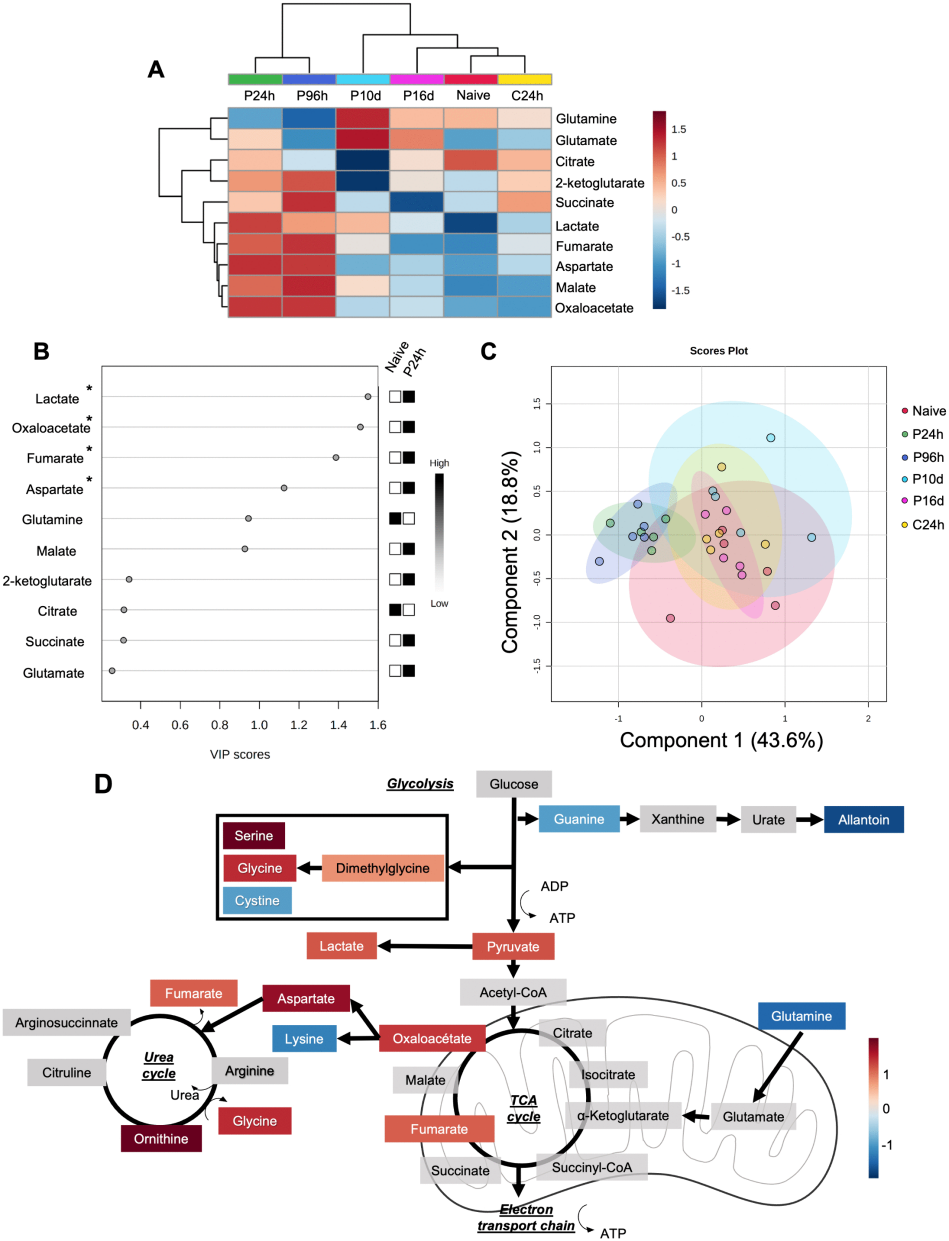
Metabolomics analyses targeting 10 specific metabolites from the TCA cycle in hemocytes. **A.)** Heat map representing in a blue and red gradient, the average quantity of each metabolite for each time points of the infection kinetic clustered by Euclidean distance. **B.)** Comparison of naive vs P24h samples, top 10 metabolites with the best VIP score (>1) (Variable Importance in the Projection) based on the PLS-DA model. Heat map with white and black boxes represent high and low abundance ratio for the corresponding metabolite in the respective sample. * Indicate VIP with significant difference (FDR < 0.05) comparing naïve to P24h. **C.)** PLS-DA analysis. PLS-DA score clustering plot for the comparison of the 10 metabolites from the different kinetic times, grouping 5 replicates per condition (colored dots). Colored circles represent 95% confidence intervals. **D.)** Schematic representation of major metabolites, related to glycolysis, TCA cycle and urea cycle, differentially represented in the P24h condition with a blue-to-red gradient representing the average quantity of each metabolite.

To validate experimentally the results obtained in our transcriptomic and epigenomic approaches (Figs 2, 4 and 5) we performed a targeted metabolomics analysis comparing hemocyte recovered from naïve, primary infected and secondary challenged snails (Figs 6 and 7). When considering the 114 targeted metabolites approach (S9 and S10 Tables), 54 specific metabolites were detected in *B. glabrata* hemocytes (Fig 6A). Analysis of these 54 metabolites shows a strong shift in metabolite accumulation in hemocytes very early after the primary infection. As shown by the heatmap and the dendrogram clustering (Fig 6A), samples that are more different from the others are P24h and P96h. Thus, the metabolism of the hemocytes changed drastically during the first 4 days after primary infection and then gradually returned to a pattern close to naïve snail hemocytes (Fig 6A). Interestingly, there is very limited changes in hemocyte metabolism following the secondary challenge. The naïve, P16d and C24h samples are grouped in the same cluster and appeared thus highly similar (Fig 6A). The primary infection induces the most significant changes in the metabolome of hemocytes while the secondary challenge did not. Significant changes (FDR < 0.05) observed from P24h were related to accumulation of metabolites belonging to amino acids (glycine, dimethylglycine and serine), of the urea pathway such as ornithine, or metabolites involved in the glycolysis pathway and the TCA cycle such as pyruvate, lactate and aspartate (Fig 6B and S10 Table). At the same time, we find a decrease in allantoin, also involved in the urea pathway, as well as some amino-acids cystine, lysine and glutamine (Fig 6B and S10 Table). Concerning the secondary challenge, when comparing C24h to P16d (S9 and S10 Tables), no significant differences could be observed (Fig 6A and 6C).

Considering that glycolysis and TCA cycle appeared as the principal metabolites accumulating following a primary infection, we performed targeted metabolomics analyses on 10 specific metabolites of the TCA cycle allowing us to have a more accurate analysis of such pathway (Fig 7; S9 Table). The samples P24h and P96h, clustered together and are the most different compared to the others (Fig 7A and S9 Table). The naïve and C24h samples are the closest (Fig 7A). This focus on TCA cycle metabolites confirms a metabolic change in hemocytes as early as 24 hours post primary infection and the absence of changes following the secondary challenge (Fig 7A and 7C). This metabolic shift is moving towards aerobic glycolysis, demonstrated by the accumulation of lactate and to the increase in TCA cycle activity with the accumulation of key metabolites like oxaloacetate, fumarate and aspartate (significant accumulating VIP, FDR < 0.05) at P24h (Fig 7B and 7D). This shift in energy production by the hemocytes lasts at least until 96 hours post primary infection and progressively returns to a basal state similar to the naïve hemocyte sample from day 10 to day 16 after primary infection (Figs 6A-6C and 7A-7C). The C24h sample clustered with the naïve and P16d samples, indeed no significant changes in VIP quantification could be observed following the secondary challenge (S9 and S10 Tables).

## Discussion

Innate immune memory in non-vertebrates is believed to rely on three main processes: (i) a recalled response, involving the activation and dampening of the immune response following a first encounter and neutralization of a pathogen by a faster and stronger activation of the same immune defence compounds in response to a secondary encounter, (ii) a sustained response, involving the long-lasting up-regulation of the same immune effectors after an initial infection; and (iii) an immune shift, involving the activation of specific gene clusters after primary infection followed by the activation of different subsets of gene clusters after a secondary immune challenge [3,10,22,32,35,36].

**Fig 8:**
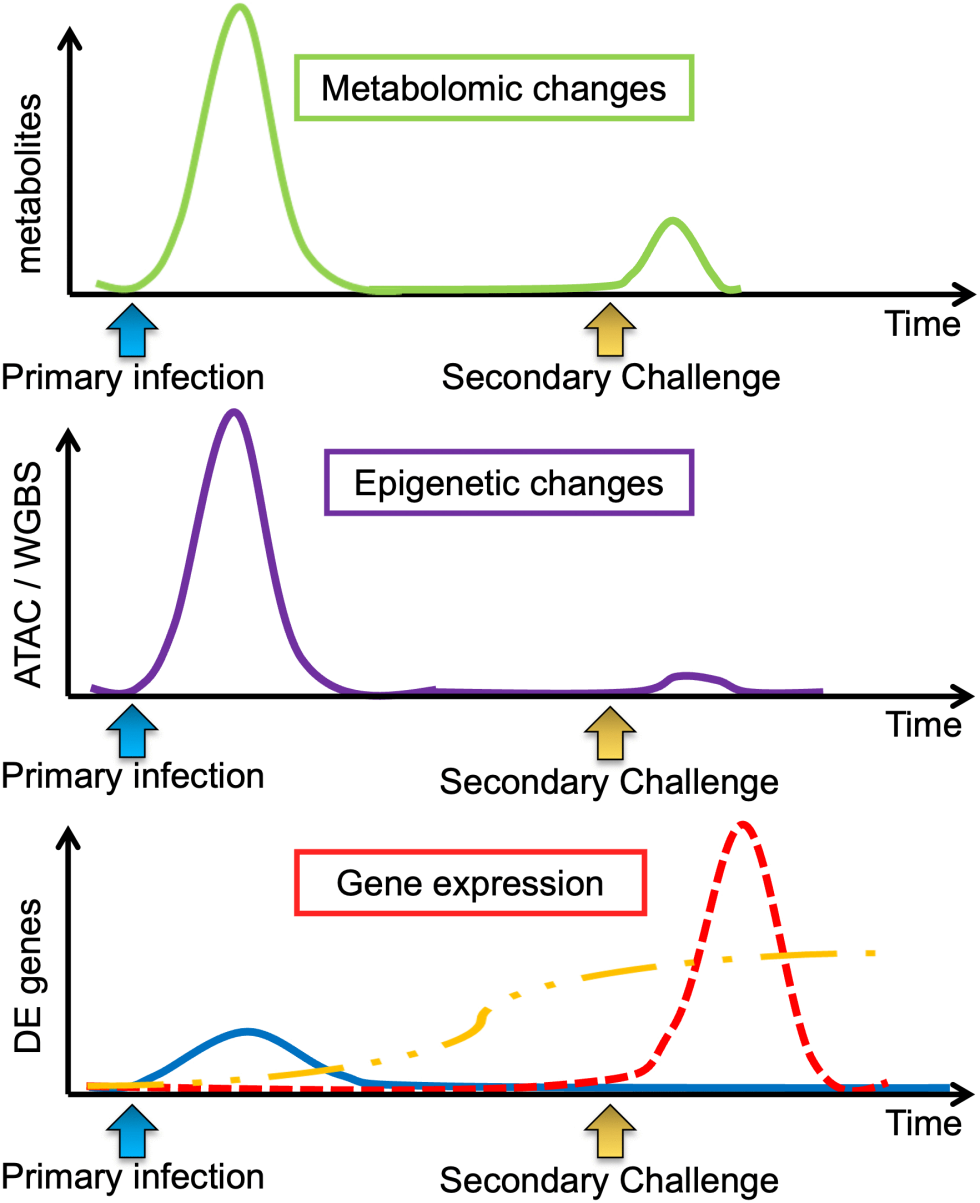
Basic models of the molecular support of innate immune memory in hemocytes of Biomphalaria glabrata. In response to a primary infection (blue arrows), metabolism (green line) and epigenetic marks (purple line) changes drastically in hemocytes, rather few changes were observed following secondary challenge (yellow arrows) and differential gene expression remained limited (blue line). However, such metabolic and epigenetic reprogramming activate a humoral immune response that may either be sustained in a long-lasting upregulation of immune effectors (broken orange line); or result in an immune shift of humoral effectors (broken red line).

Herein, we analyse those components of the innate immune memory response and their regulation processes exclusively for the hemocytes, the innate immune cells of *Biomphalaria* snails exposed to primary infection and secondary challenge with its natural parasite *Schistosoma mansoni*. Based on the present RNAseq results, we were able to confirm, as previously observed in whole snails [11,18,21], that the cellular immune response activated after the primary infection is supported by a limited subset of hundreds of DEGs, compared to the thousands of genes that are activated after the secondary challenge (Figs 2 and 8). Among these DE genes, 6.6% exhibited a “recalled pattern”, 28.5% a “sustained response” pattern and 64.9% were specifically associated with the “immune-shift” response. The immune-shift is particularly marked in hemocytes, much more so than what it was in whole snails [18], demonstrating, as a first important result, that hemocytes may likely be the main cells carrying innate immune memory (IIM) in *Biomphalaria glabrata* snails.

Based on those results we can conclude that IIM is supported by both, a sustained response and a shift response, but those responses appeared very different in their respective roles in the defence of the organism. Induction of the response as early as P16d with a generalist response activated from the primary infection (sustained) could explain the anticipatory response that enhances the immune capacities of hemocytes while also promoting their training and reprogramming. This training likely contributes to the immune shift, which is activated exclusively following the secondary challenge. Using enrichment analysis, we were able to identify specific pathways differentially represented in sustained and shift clusters (S4 Table). The sustained response is associated with functions related to membrane receptors, cell proliferation/ migration/ differentiation and cell metabolism, that could be clearly involved in the hemocyte reprogramming, preparing the cells for acquiring new capabilities and functions. The immune shift response is associated with cell metabolism (urea cycle/ TCA/ glucose/ OxPhos), regulation of transcription and self/non-self-recognition (pattern recognition receptors (PRRs)) that can support the high specificity of this immune response [21] and the huge transcriptional response associated with, which certainly requires a very strong energy production in these cells following the secondary challenge. As previously hypothesized [18,21], we confirm in the present study that most of the genes differentially regulated at 6h and 12h after the secondary challenge belong especially to a humoral response (Fig 3 and S3 Table). Interestingly, within those early activated genes after the secondary challenge, some have been shown in the literature to be involved in anti-schistosome response in snails: Fibrinogen related proteins (FREP, belonging to hypervariable pattern recognition receptors) [37–41], Thioester containing protein (TEP, belonging to complement pathway) [37,42–45] and Biomphalysin (belonging to beta pore forming toxin family) [39,46–48]. Recent results suggest a scenario in which *Bg*FREP and *Bg*TEP could be the factors associating with Biomphalysin to promote plasma-mediated *S. mansoni* sporocyst killing in *B. glabrata*. Indeed, this immune protein complex would be necessary to activate the oligomerization of Biomphalysin toxins resulting in a functional transmembrane pore that kill sporocyts [49]. Such mechanism being potentially similar to the membrane attack complex described in the lectin pathway of the complement system [50,51]. This immune complex driven by FREP molecules is also particularly relevant considering the high level of specificity of IIM in *Biomphalaria glabrata* snail [11,18,21]. It was demonstrated that *B. glabrata* regulates a polymorphic set of FREP variant repertoires to respond to different strains of Schistosoma parasites following a secondary homologous or heterologous challenge. These results suggest a combinatorial usage of pathogen recognition receptors (PRRs) that distinguish different strains of parasites during the acquisition of immunological memory [21].

Moreover, the persistence of IIM throughout snail life [21] despite the rather short lifespan of the hemocyte cells in the hemolymph, suggests that the memory of past infections could reside in the hemocyte precursors, such as hematopoietic stem cells present in the hematopoietic organ. However, identifying the specific tissue in which the immune memory is stored and appropriately reactivated when needed deserves further investigations. Herein, we observe that the same proportion of each hemocyte subpopulation remained stable even after IIM acquisition (Fig 2B). The bulk-hemocyte RNAseq approach conducted herein give us relevant results about hemocyte response during IIM process, but was not appropriate to identify a specific population of hemocyte that would be supporting the recording of the encounter with the pathogen. Recently, we demonstrated using a single-cell-RNAseq approach (scRNAseq) that the hemocyte heterogeneity in *Biomphalaria glabrata* naive snails seems to be more related to transcriptional plasticity rather to morphological diversity [52]. If we identify 3–4 morphologically distinct hemocyte populations, 7 hemocyte transcriptomic populations were defined, revealing a great hemocytes transcriptional plasticity with a probable capacity to engage into different activation pathways. Thus, to go further in the investigation of the hemocyte role in IIM, a particular attention would have to be paid for individual cells expression patterns that might become more numerous after priming. In a near future, the differences between hemocytes would have to be thus investigated at the transcriptional level using scRNAseq approaches.

Concerning the epigenetic and metabolomic processes in hemocytes, the main changes were observed following the primary infection and almost no changes were observed following the secondary challenge (Fig 8). This observation is particularly relevant considering that hemocytes must acquire new capabilities and that the acquisition of IIM could be linked to these changes in the epigenetic landscape and metabolism of hemocytes. When this metabolic and epigenetic reprogramming is stabilized in the hemocytes and IIM acquired (taking around 10 days, [20]) then no new changes occur in term of metabolism or epigenetic even after a new encounter with the same pathogen following the secondary challenge (Fig 8).

While many studies have examined the epigenetic based events that support IIM in the mammalian context, little is known about how these ideas may extend to non-vertebrates [53]. Most of the work done until now on this question was dedicated to insects. Evidence exists that DNA/RNA or histone epigenetic marks (methylation or acetylation) can participate in the activation of the primary innate immune response following the first exposure to a pathogen [3,53,54]. For example, DNA methyl transferase (DNMT) expression changes following bacterial infection for the insects *Helicoverpa armigera*, *Drosophila melanogaster* or *Aedes aegypti* [55–57]. Changes in histone acetyltransferases (HATs) and histone deacetylases (HDACs) activities were observed in *Galleria mellonella* after infection with entomopathogenic fungus [58]. Similarly, HAT regulates the production of hemocyte differentiation factor (HDF), which regulates the cellular immune response of *Anopheles gambiae* against *Plasmodium* [59]. Bacterial or fungal infections in *Tenebrio molitor* were associated with reduced RNA methylation [60].

To our knowledge, most studies investigating epigenetic regulation in invertebrates following infection have focused on the expression of epigenetic enzymes or pathways, rather than on direct measurements of epigenetic marks. As a result, evidence for the involvement of epigenetic modifications in immune priming remains indirect, and the underlying mechanisms are still poorly understood [60,61].

Given this lack of direct evidence linking epigenetic modifications to immune priming, we investigate the impact of epigenetic pathways through analyzing differentially methylated regions and chromatin accessibility between naïve, primary infected and secondary challenged snails. When we look at the functions of the genes that were associated with epigenetic changes, (i) changes in chromatin accessibility (ATACseq: opening or closing chromatin regions) or (ii) hypo/ hypermethylation status (WGBS), enriched GO functions were well correlated with the GO functions identified in the RNAseq approach. In opening chromatin region of ATACseq we identify functions related to cell metabolism, immunity, cell differentiation/morphogenesis and regulation of transcription. GO functions associated with WGBS relate to cell morphogenesis/ development/ activation, inflammatory response, mitochondria regulatory process or epigenetic regulation. Interestingly, ATACseq opening regions shared 35.6% of its genes with the sustained response profile and 21.6% with the immune shift response (S3 and S7 Table). Thus, increasing chromatin accessibility better supports the sustained immune response than the shift response in IIM.

Concerning DNA methylation for non-vertebrates, the role and function of DNA methylation appeared as a complex black box. The detailed molecular mechanisms through which DNA methylation signaling contributes to regulate gene expression are yet to be explored. DNA methylation has been demonstrated as playing a role in protecting animal genomes against the harmful effects of transposon activity or to environmental stressors, but the link with transcriptional regulation remains weak or unclear [62–65].

DNA methylation may be also implicated in genome stability and environmental responses. Experimental alteration of methylation patterns in Biomphalaria glabrata has been shown to affect phenotypic traits and gene function [66]. Moreover, components of the DNA methylation machinery are modulated during host–parasite interactions, suggesting a role in the response to *Schistosoma mansoni* infection [67]. Consistent with this, we did not observe a clear correlation between differentially methylated genes and transcriptional changes in *Biomphalaria glabrata*. Only a limited proportion of differentially methylated genes are correlated with differentially expressed genes, with heterogeneous expression patterns. Nevertheless, differentially methylated genes identified in this study were associated with key signaling pathways involved in immune cell activation, suggesting that DNA methylation may contribute to the regulation of hemocyte functional states. Altogether, these findings support the idea that DNA methylation does not act as a direct driver of transcriptional changes but may instead participate in the priming or stabilization of immune responses, potentially contributing to innate immune memory. For ATACseq, opening and closing of chromatin regions were more directly related to gene expression. However, how such changes in chromatin structure are regulated remained a complex question in *Biomphalaria* snail due to the lack of tools for histone mark modification analyses. The overall molecular mechanisms underlying epigenetic modifications in *Biomphalaria* remain to be elucidated.

Thus, the epigenetic marks analyzed in the present study were perhaps not the only ones that we needed in order to decrypt the epigenetic support of IIM in *B. glabrata* snails. For example, miRNA or LncRNA are known to greatly influence the regulation of gene expression [68–70] and histone marks (acetylation or methylation) are also of central importance in the acquisition of immunological memory [71]. Unfortunately, CHIPseq, which is the best approach for analyzing histone mark modifications, is not yet available for *Biomphalaria* snails.

Epigenetic and metabolic changes coexist in vertebrates, cellular metabolism provides the substrates necessary for epigenetic modifications, and this metabolic and epigenetic reprogramming is a central feature of trained immunity in vertebrates [59,72]. In non-vertebrates the question is seldom studied. In insects and more especially in mosquitoes it has been demonstrated that infection triggers metabolic changes, such as carbohydrate metabolism, TCA cycle, lipid metabolism, and fatty acid synthesis during activation of immune memory [73]. More direct evidence were obtained from the crustacean *Eriocheir sinensis* for which changes in histone methylation marks (H3K9me3) were demonstrated in the promotor region of key enzymes of the glycolysis and of the pentose phosphate pathways in hemocytes following a secondary challenge with the bacterium *Aeromonas hydrophila* [74,75].

Based on those previously exposed results, cell metabolism appears as a central pillar of IIM acquisition. In the present paper, metabolic GO functions are always identified in the different omics approaches conducted and more precisely cell metabolism related to TCA cycle, Amino acid synthesis, glycolysis, urea cycle and oxidative phosphorylation (OxPhos) are particularly recurrent in RNAseq, ATACseq and WGBS. Thus, to validate such metabolic modifications in hemocytes, we conducted a targeted metabolomic approach. We were able to describe critical changes in metabolism of hemocytes very early following primary infection with (i) an increase of the glycolysis pathway that is demonstrated by the accumulation of pyruvate and lactate in hemocytes; (ii) modifications in the urea cycle and urate metabolism with the increased accumulation of ornithine and the decrease of allantoin; (iii) amino acid synthesis pathway with increased proportion of glycine and serine and a decrease of cystine and lysin; and (iv) truncated tricarboxylic acid (TCA) cycle due to the activation of glycolysis that is demonstrated by the decrease in glutamine due to the glutaminolysis directly linked to the replenishment of the TCA cycle that results in the accumulation of aspartate, oxaloacetate and fumarate which are parts of TCA cycle metabolites.

The objective of this integrative approach is to join all the results based mainly on similarity between enriched pathway in each specific approach and look at the correlation of molecular pathways that were shared between each level of epigenetic, metabolic and transcriptomic processes investigated. Thus, we believe that following infection by a pathogen, a competent subpopulation of hemocytes could be activated through multistep events of differentiation and reprogramming, involving metabolic, epigenetic and transcriptomic regulatory processes to enhance hemocyte responses and immune memory capacities. In response to a primary infection, hemocyte metabolism and epigenetic marks changed drastically, whereas few changes were observed following secondary challenge (Fig 8). Such metabolic and epigenetic reprogramming activate IIM in hemocytes and prepare the cells to achieve the huge transcriptomic response towards pathogens that may either be sustained in a long-lasting upregulation of the immune response or resulted in the immune shift of more specific humoral effectors.

It is important to note that, many genes that are differentially regulated, or associated with epigenetic changes and metabolites differentially represented in hemocytes during the process of IMM acquisition are mainly involved in multiple interesting pathways and functions already described in vertebrate trained immunity like glycolysis, pentose phosphate, TCA cycle pathway, urea pathway, lipid metabolism or mitochondrial OxPhos [76–79]. Indeed, innate immune cells of vertebrates can adopt long-term inflammatory phenotypes following encounters with a pathogen, this phenomenon is called trained immunity and can improve host defence against (recurrent) infections. The mechanisms supporting trained immunity in vertebrates principally include profound changes in intracellular metabolism, which are closely associated with epigenetic reprogramming. Moreover, glycolysis and glutamine replenishment of the tricarboxylic acid cycle (TCA) with accumulation of fumarate, and the mevalonate pathway have all been identified as critical pathways for trained immunity [78]. Briefly, trained immunity activation is mediated by changes in cell metabolic pathways that result in epigenetic rewiring of cellular functional programs. Glycolysis, and glutaminolysis pathway are indispensables for the induction of trained immunity. Accumulation of fumarate, due to glutamine replenishment of the TCA cycle, induce monocyte epigenetic reprogramming by inhibiting KDM5 histone demethylases and fumarate itself can induce epigenetic changes in immune cells [72,80]. Intermediates from glucose catabolism during glycolysis can branch out through the serine one‐carbon metabolism to generate amino acids of serine and glycine fuelling into the folate and methionine cycle to generate *S*‐adenosyl‐methionine (SAM) [81]. SAM might drive metabolic and epigenetic reprogramming by histone and DNA methylation which plays a central role in chromatin dynamics and gene expression patterns [82]. Shift in energy production in innate immune cells is known as Warburg effect which is one such signature of how glycolysis influences metabolic shift during trained immunity. In cells, Warburg effect results in the repression of energy production through TCA cycle and oxidative phosphorylation in the mitochondria and enhances the inducible process of ‘aerobic glycolysis’ consisting of high level of glucose uptake and glycolysis followed by lactate fermentation taking place in the cytosol [78].

During IIM acquisition, following primary infection and secondary challenge, similar molecular processes were observed in *Biomphalaria* snails. Hemocytes appear to undergo metabolic reprogramming, likely to meet the energetic demands associated with immune activation, including transcription, differentiation, and proliferation [82,83]. In particular, we observed a shift towards aerobic glycolysis, glutaminolysis, and a reduction in oxidative phosphorylation, consistent with a Warburg-like metabolic profile.

These metabolic changes, at 24h and 96h after the primary infection, are associated with the accumulation of key metabolites, such as fumarate and other TCA cycle intermediates, which have been shown in vertebrate systems to influence epigenetic enzymes, chromatin modifications and acquisition of trained immunity [72,83]. In addition, alterations in amino acid metabolism and the urea cycle were observed, pathways that have also been implicated in shaping immune cell function and epigenetic states in vertebrate systems and induce innate immune memory [84–86]. Finally, the insulin signaling pathway was also modified in hemocytes, and has been implicated in the regulation of immune cell activation and inflammatory responses [87].

While direct evidence linking these metabolic changes to epigenetic reprogramming in Biomphalaria is still lacking, our results suggest that similar mechanisms may be involved. Together, these observations point to a coordinated metabolic and transcriptional reprogramming of hemocytes during IIM acquisition, potentially contributing to enhanced immune response upon secondary challenge. Through pathway-level integration of transcriptomic, epigenomic and metabolomic data, we identified central molecular pathways that are consistently associated with the acquisition of innate immune memory in *Biomphalaria* snail hemocytes. Interestingly, similar pathways have been described in the context of trained immunity in vertebrate innate immune cells. Based on these observations, our results suggest that comparable mechanisms, i.e., “trained immunity”, may operate in *Biomphalaria glabrata* [2]. More broadly, the presence of shared molecular features across different animal lineages supports the hypothesis that trained immunity may represent an evolutionarily conserved trait as hypothesized in previous opinion papers [2,26]. However, our data remain correlative, and further functional studies will be required to determine the extent to which these pathways are directly involved in the induction of innate immune memory in this system

However, it may not be reasonable to believe that acquired immunity or innate immune memory are a general characteristic of the entire living kingdom [2,5,77,88]. Unifying or generalising these mechanisms to all non-vertebrate phyla would be an oversimplification that could mask the great complexity of immune mechanisms, which can generally differ in these biological systems. Therefore, it is now needed to obtain an overview of this new concept and understand the nature and origin of these conserved characteristics by analysing the molecular basis of innate immune memory in a growing number of phyla of the animal kingdom, taking into account the diversity of the interactions studied.

## Materials and methods

### Ethics statement

Our laboratory holds permit # A66040 for experiments on animals from both the French Ministry of Agriculture and Fisheries, and the French Ministry of National Education, Research, and Technology. The housing, breeding and animal care of the utilized animals followed the ethical requirements of our country. The researchers also possess an official certificate for animal experimentation from both French ministries (Decree # 87–848, October 19, 1987). Animal experimentation followed the guidelines of the French CNRS. The different protocols used in this study had been approved by the French veterinary agency from the DRAAF Languedoc-Roussillon (Direction Régionale de l’Alimentation, de l’Agriculture et de la Forêt), Montpellier, France (authorization # 007083) and the Ethic committee CEEA-LR (# C66-136–01).

### Biological materials, experimental approaches and infection procedures

The Brazilian strain of *Biomphalaria glabrata (*BgBRE2, locality of Recife) was used for all the experimental approaches conducted in the present study. Parasite infections were conducted using the 100% compatible homopatric parasite strain of *Schistosoma mansoni* originating from Brazil (SmBRE, locality of Recife). SmBRE was maintained in its homopatric BgBRE2, and in mice (Swiss OF1), as described previously [11,89]*.* Briefly, miracidia from SmBRE were hatched from eggs axenically recovered from 60-days infected mouse livers. Livers were collected and homogenized, and the eggs were filtered and washed to obtain miracidia [90].

BgBRE2 Snails (aged of 3–4 months, size between 7–9mm) were individually primary infected with 10 miracidia of SmBRE (noted “P” for Primary infected). Experimental infections were conducted by infecting BgBRE2 snail individually in wells containing 2 mL of spring water with 10 miracidia of SmBRE and letting for 3h for natural infection to occur. Naïve snail used in the following described experiments were also treated in the same way but without putting the parasites. Fifteen days after primary infection, snails confirmed as infected (observation across the shell of SpII in the digestive gland, (Fig 1)) were individually secondary challenged with 10 miracidia of SmBRE (noted “C” for secondary Challenged). During the experiment, different hemocyte samplings were performed in naïve, primary infected and secondary challenged snails at different time points to perform molecular analyses as explained in Table 1 and Fig 1. Hemocytes were recovered by foot puncture of hemolymph according to an established procedure [91]. Hemolymph was then spin-down (500 g, 10 min, 26°C) to pellet the hemocytes. Hemocytes were sampled in a time kinetic from naïve snails, at 6 hours, 12h, 24h, 4 days, 10 days, 15 days and 16 days after primary infection (samples were named naive, P6h, P12h, P24h, P96h, P10d, P15d, P16d), and at 6 hours, 12 hours and 24 hours after the secondary challenge (samples were named C6h, C12h, C24h) (Fig 1). Naive snail samples were recovered 24h after the primary infection and P16 samples 24h after the secondary challenge. For the number of snails used and the number of replicates see Table 1. For all the experiments conducted herein, the status of the infection was assessed by measuring its prevalence. Briefly, snails (after hemolymph sampling) were fixed in Raillet fixative solution (6g/L NaCl, 20 ml/L Acetic Acid, 50 ml/L formaldehyde 37%) during 48 hours before being dissected under a stereo-microscope (Fig 1) to detect and count the primary and/or secondary sporocysts [92]. A mollusc was confirmed as infected if at least one primary and/or one secondary sporocyst was identified. Prevalence percentage was calculated as follow: P = (number of infected snails/ total number of exposed snails) * 100.

### Total hemocyte count and proportion of hemocyte populations

Total hemocyte counts were estimated on 37 BgBRE2 snails for naive, P24h, P16d and C24h time points, and individually enumerated (number of hemocytes/ µl of hemolymph) using a malassez chamber. Hemocyte population counts were quantified on 10 snails for naive, P24h, P16d and C24h time points (see raw counts on S1 Table). For hemocyte population counts, the hemolymph was poured on a polystyrene slide and left in a humid chamber for 30 min to allow hemocytes to adhere to the slide. Hemocytes were then stained using MCDh (RAL Diagnostics) protocol (6’ in MCDh1, 1’ in MCDh2, 2’ in MCDh2, 1’ in MCDh3, 10” in MCDh4), then slides were dried and mounted with Dako fluorescent mounting medium (Agilent, S3023) and the hemocyte populations (hyalinocytes, granulocytes, blast-like cell) are distinguished and counted under light microscope. Significant differences in hemocyte counts were estimated using a Kruskal-Wallis rank sum test associated with Dunn post-hoc test further adjusted by the Benjamini-Hochberg FDR method.

### RNAseq approach: mRNA recovery and sequencing

Pools of hemolymph recovered from 12 BgBRE2 snails were sampled at 6, 12, 24, 96 hours and 16 days after primary infection; these samples were designated P6h, P12h, P24h, P96h and P16d, respectively. Similar pools were recovered at 6, 12 and 24 hours after the secondary challenge and named C6h, C12h, C24h respectively (Fig 1 and Table 1). All these samplings were done in triplicate. Triplicate pools of hemolymph recovered from 12 uninfected snails were used as control (naïve hemocyte samples). All hemolymph samples were centrifuged at 2000g, 5 min at 26°C to pellet the hemocytes before RNA extraction. Trizol reagent (Sigma Life Science, St. Louis Missouri, USA) was used to extract total RNA regarding the manufacturer’s guidelines. The quality of extraction was assessed by Bioanalyser profiling before sending the samples to be sequencing. Library preparation (Stranded mRNA, Illumina) and HiSeq 3000 (Illumina) pair-end sequencing (2x75pb) were performed by Fasteris platform (Plan-les-Ouates, Switzerland).

### RNAseq data processing

To perform the differential expression analyses, the Galaxy platform has been used [93]. Briefly, read quality was checked using Fastqc tool [94] and adapters were trimmed by Trim Galore Tool version 0.4.3.1 [95]. High quality and adaptor-free reads (phred>28) from each library were mapped on the annotated *Biomphalaria glabrata* genome version 1.7 from VectorBase [96], with RNA STAR version 2.6.0b-2 [97]. Htseq-count version 0.9.1 [98] was used to estimate the number of reads mapping to all genes. Finally, DESeq2 version 2.11.40.2 [99] was used to calculate differential expression (DE) of all transcripts during the kinetic compared to the control samples. The result consists of a list of DE genes compared to control (naïve snails) with log2 fold change (log2FC) value superior to 1 and inferior to -1 (-1 > log2FC>1)) and padj value < 0.01 for each gene and for all the eight time-points of the infection kinetic. Another analysis was also conducted to compare the DE genes between secondary challenged samples (C6h, C12h and C24h) and the P16d sample used as control for the secondary challenge infection. For all the tested conditions and biological replicates, the sequencing depth remains relatively homogeneous, with an average of 40,552,291 reads per sample that mapped to the genome (S2 Table).

### RNAseq enrichment analysis

Heat map of DE genes was created with Treeview3 [100]. A hierarchical clustering classification is used; similarity metric is calculated using Euclidean distance and a single linkage was used as the linkage method for clustering. This heat map helps us to determine and classify all genes according to their DE pattern, which was followed by an enrichment analysis for gene clusters of interest. The enrichment analysis was performed with Blast2GO version 5 [101]. The annotation in GO terms of each gene was taken from VectorBase [96]. For each GO term, an enrichment test based on the Fisher’s exact test was applied.

Only enhanced functions with a P-value < 0.01 were kept in all three Gene Ontology (GO) classifications: biological process, cellular component and molecular function. A list of over- and under-represented functions in our samples was then created. A diagram representation of enriched GO terms was produced using the Revigo software [102], using GO IDs and associated P-values. Finally, Venny 2.1 [103] was used to create Venn diagrams for analyzing the number of GO functions shared by different conditions, especially for the secondary challenge time-points. Similar enrichment analyses have been conducted for WGBS and ATACseq approaches. For WGBS hyper-methylated and hypo-methylated genes were analysed separately. For ATACseq open and closing chromatin regions were analysed separately.

### ATACseq approach: DNA recovery and sequencing

For the ATACseq (Assay for Transposase-Accessible Chromatin with high-throughput sequencing) experiment, seventy BgBRE2 snails were used. Twenty snails were used as control, while fifty snails were individually exposed to 10 miracidia of SmBRE; and 25 snails were secondarily challenged 15 days later with 10 homologous miracidia of SmBRE. Hemolymphs from 2 pools of 10 snails were recovered for each experimental sample (naive, 24 hours after primary infection or secondary challenge snails called Naïve, P24h and C24h respectively). Around 25 µL of hemolymph were recovered from each snail with around 300 cells per µL. Each experiment was thus performed on ±80,000 cells. Hemolymph was centrifuged 2000 x g for 5 min at 26°C to pellet cells. Cells were washed with cold CBSS buffer [37] then centrifuged at 500 x g for 5 min at 4°C. Nuclei were extracted from harvested cells as previously described [104]. Briefly, pellets of cells were gently re-suspended in 500µL of cold lysis buffer (10 mM Tris-HCl, pH = 7.4, 10 mM NaCl, 3 mM MgCl_2_, 0.1% IGEPAL CA-630) before spinning down immediately at 500 x g for 10 min at 4°C. Pellets of nuclei were immediately submitted to a transposition reaction adapted from previously described procedures: 25 µl 2x TD Buffer (Illumina), 1 µL Tn5 Transposase (Illumina) and 24 µL Nuclease Free water, for 20 min at 37°C. DNA was purified on Qiagen MinElute Kit following manufacturer guidelines (Qiagen, Hilden, Germany). DNA fragments were amplified in order to constitute sequencing libraries. Briefly, DNA was amplified using NEBNext High-fidelity enzyme (New England BioLab, Ipswich Massachusetts, USA) and Customized Nextera PCR Primers that allow to differentiate libraries during multiplexed sequencing. The number of optimal cycles was estimated thanks to qPCR experiments as previously described [104]. The selected PCR cycles were (1) 72°C 5mn, (2) 98°C 30s, (3) 98° 10s, (4) 63°C 30s, (5) 72°C 1mn, steps 3–5 were repeat 10 times to complete exactly 11 cycles per library. Amplified libraries were purified using Qiagen PCR Cleanup kit (Qiagen, Hilden, Germany), checked on electrophoresis BioAnalyser instrument (Agilent) with DNA 1000 kit before being sent for HiSeq 2000 sequencing (Illumina) in 2x75 pair-end by the Fasteris platform (Plan-les-Ouates, Switzerland).

### ATACseq data processing

Quality control was performed on FASTQ files with the Fastx-toolkit suite. No trimming was necessary as all files passed our criteria showing average quality values per base above 30. The alignment was performed with Bowtie2 using -X 2000 and default parameters. We used Macs2 callpeak to identify peaks using the following parameters: -f BAM –nomodel –shift -75 –ext size 150 -B. The resulting BedGraph file was used to call the peaks using macs2 bdgpeakcall with parameter -l 100 and -g 25. Afterwards, Bedtools multicov was used to produce the count file with parameter -f 0.5. The count file was used to identify differentially open peaks using DESeq2 on a local Galaxy server (https://bioinfo.univ-perp.fr/). The intersection between open chromatin and differentially expressed genes was done using Bedtools intersect with parameter -u to obtain each gene only once.

### WGBS approach: DNA recovery, bisulfite conversion and sequencing

450 BgBRE2 snails were infected by 10 miracidia of *S. mansoni* and 450 naïve snails were used as control. Fifteen days after primary infection, the hemolymph of the 450 infected snails and 450 naïve snails was recovered by foot puncture. 3ml of hemolymph from three pool of 150 snails (biological replicates) were used for DNA extraction for each treatment. Samples were named according to the following nomenclature: Naïve 1, 2, 3 stands for *B. glabrata* controls, P15d-1, 2, 3 stands for *B. glabrata* infected with *S. mansoni* for 15 days. Genomic DNA was extracted from 3ml of pooled hemolymph samples in triplicate for each condition (infected and control). For that, samples were put in 1 ml of lysis buffer (20 mM TRIS pH 8; 1 mM EDTA; 100 mM NaCl; 0.5% SDS), with 0.3 mg of proteinase K at 55°C for 1 night. Then, DNA was purified by two rounds of phenol-chloroform extraction, precipitated as previously described [105] and dissolved in 40μl water. DNA concentrations were measured with QuBit 2.0 analyser (Invitrogen) using the broad range kit according to manufacturer recommendation. 2.3 μg of total gDNA were sent to the sequencing facility except for one sample (Naive3) for which we sent only 1.7μg which was the maximum amount that we had extracted (see S4 Table for details). The Genome Quebec sequencing platform performed bisulfite conversion and WGBS paired-end library construction and sequencing, which was performed on a Hiseq4000 using 125 nucleotide paired-end reads.

### WGBS data processing and differentially methylated gene identification

Quality metrics are indicated in S2 Table. Read quality was checked using FastQC. Nucleotide positions with a quality score below 20 and sequencing adapters were trimmed using Trimgalore (paired-end, both strands, phred quality score = 20). Reads were then aligned on the *B. glabrata* reference genome (*B glabrata* genome version 1.7 from VectorBase [23] with Bismark [106] using the following parameters: maximum insert size = 500, no mismatch allowed, sort Bam file by chromosomal position, other parameters used as default). Clustering of the data was performed from BAM files using MethylKit [107]. And using MethylExtract [108] and the BAM files from Bismark, the methylation level of each cytosine was synthesized in different file formats (Bed, Wig and VCF). The Wig files were loaded on Integrative Genomics Viewer (IGV) (https://software.broadinstitute.org/software/igv/) for visual inspection of the data [109]. Differential methylation analyses were performed with DMRseq package using the following parameters blocksize = TRUE, minnumregion = 3, deltamax = 0.25, bpspan = 1000, mininspan = 10, maxgapssmooth = 2500, smooth = TRUE [110] as previously optimized in the lab for differential methylation analysis on other mollusk species. The R scripts used here can be downloaded at https://github.com/IHPE/DMRseq_wrapper. DMRs were intersected with the annotation of *B. glabrata* genome (version 1.7 from VectorBase) to identify DMRs that occurred within genes (Differentially Methylated Genes (DMGs)) and within promoters (Differentially Methylated Promoters (DMPs)). The + 2kb region upstream of the transcription start site was defined as the promoter region. DMGs were used for functional annotation and enrichment analysis with GO enrichment analysis.

### Metabolomics approach: Sample recovery, metabolite extraction

All mollusks (BgBRE2) were maintained in common garden in a 60 L tank with perforated partitions walls allowing water to circulate between 3 compartments of 20 L. Each compartment permits to separate, the uninfected snails (naive) from the primary infected and the secondary challenged snails. Before the snails were collected, tank-water was replaced and food was removed (24h starvation). Then, hemolymph was recovered for control (naïve snails) and at 1 day, 4 days, 10 days, 16 days after primary infection, and 1 day after the secondary challenge. The numbers of hemocytes per sample were counted in Malassez chamber and a volume of hemolymph corresponding to 60 000 hemocytes was recovered. For each time point of the kinetics, 5 biological replicates (each corresponding to a pool of 20 snails) were sampled. The hemocytes were centrifuged (2700 x g, 5 min, 4°C), washed once with 500µl of CBSS (Chernin’s balanced salt solution), then 500 µl of acetonitrile were added to the hemocyte pellet and sonicated (3 pulses of 10 secs at 20% amplitude). The samples were then stored at -80°C until used. To confirm IIM acquisition, prevalence and intensity were measured for 45 snails recovered after primary infection and secondary challenge. For this purpose, the snails were removed from their shells and stored in Raillet’s fixative solution for at least 48 hours prior to dissection.

### Metabolomics data processing

Sonicated hemocyte samples (5 replicates of 60 000 hemocytes per kinetic time points) were crushed using glass beads (Dominique Dutscher, Issy-les-Moulineaux, France) and Precellys Evolution homogenizer (Bertin Instruments, Montigny-le-Bretonneux, France) in a mix of 600 μL of lysis buffer containing 80% methanol (Sigma Aldrich), 20% water and internal standard (IS) 2-morpholinoenthanesulfonic acid (10 μM final concentration, Dojindo, Tokyo, Japan). Following crushing, the sample was first vortexed vigorously followed by centrifugation at 15 000 x g for 15 min/4°C. The clear upper phase was removed and filtrated through Captiva ND Lipids 96-well plate (Agilent Technologies, Santa Clara, CA, USA), to remove proteins and lipids from the sample. The filtrate was then lyophilized and subsequently dissolved in 50 μL of ultrapure water. Three microliters of prepared sample were injected on UPLC coupled to triple-quadrupole MS (LCMS-8050; Shimadzu, Kyoto, Japan). The chromatographic conditions were as follows: analytical column Discovery HS F5-3 (Sigma Aldrich), mobile phase A 0.1% formic acid in water (Wako), mobile phase B 0.1% formic acid in acetonitrile (Wako), flow rate 0.25 mL/min, column oven 40°C. The gradient was: 0 – 2 min 100% A, 5 min 75% A, 11 min 65% A, 15 – 20 min 5% A, 20.1 – 25 min 100% A. MS setting, data acquisition and data analysis were performed according to manufacturer instructions for analyzing Primary Metabolites version 2.0 (Shimadzu).

Following the data acquisition, peak surface area for all identified compounds was calculated and the data were normalized using the internal standard. Missing values for individual metabolites were imputed by assigning the least possible peak area found in the dataset. The imputation was based on the hypothesis that as long as a given metabolite was present in some of the samples, its absence in other samples was due to the limit of detection of the analysis. The data were then imported in R (4.0.2) [111]. Metabolites with inconsistent values, as estimated by visual inspection of boxplots (large deviations from median value evidenced by upper and lower quantiles), were removed from dataset. Outliers among the samples were identified using the Grubbs’s test (R package “outliers”) and if applicable were removed from analysis. The data where further analyzed using the MetaboAnalyst (v 5.0, https://www.metaboanalyst.ca/) [112,113]. Following the creation of the MetaboAnalyst object, data are scaled by Pareto scaling and transformed by generalized logarithm (glog) [114,115]. Partial Least Square-Discriminant Analysis (PLS-DA) is used to determine the variables of importance in projection (VIP) of each metabolite for all samples. Metabolites with a P-value < 0.05 (Student’s T test) and VIP value > 1 are considered significant between groups.

## Supporting information

S1 Table Hemocyte counts in infection kinectic. Total circulating hemocyte counts and total percentage of hemocyte subpopulation counts.
(XLSX)

S2 Table Sequencing quality metrics. RNAseq and WGBS quality metrics for all biological replicates.
(XLSX)

S3 Table RNAseq differentially regulated genes. Log2fold change of all significant (Pvalue < 0.05) differentially regulated genes indicated by their accession number for all infection kinetic time points. Gene annotation, GO identification number and GO name were indicated for each gene.
(XLSX)

S4 Table RNAseq enriched Gene Ontology functions. (XLSX)

S5 Table Whole Genome Bisulfite Sequencing differentially methylated regions and differentially methylated genes. Hypo and Hyper methylated regions and hypo and hyper methylated genes were indicated for significant Pvalue < 0.05 data.
(XLSX)

S6 Table Whole Genome Bisulfite Sequencing enriched Gene Ontology functions. (XLSX)

S7 Table ATACseq chromatine accessibility data. Opening and closing genomic regions in the infection kinetic.
(XLSX)

S8 Table ATACseq enriched Gene Ontology functions. (XLSX)

S9 Table Metabolomics data. Raw data of the global analyses (114 metabolites) and the specific analyses of the TCA cycle.
(XLSX)

S10 Table Metabolomics statistics. Representing for each sheet the differential expression of metabolites significantly different for conditions P1d, P4d, P10d and P16d when these conditions are compared to the naive condition. The last sheet corresponds to the same analyses for C1d but compared to its specific control P16d. The corresponding volcano plots are added next to the tables and represent -Log10 of the P-value of each metabolite as a function of log2FC. Metabolites marked in red are overrepresented in the test condition (compared to the naïve condition for post primo infestation times from 1 to 16, and compared to the P16d condition for C1d time), in blue underrepresented and in grey not significant.
(XLSX)

S11 Table Correlations between differentially expressed genes and differentially methylated genes (hypo or hyper-methylated genes). (XLSX)

S12 Table Correlations between differentially expressed genes and opening or closing chromatin region (ATACseq data). (XLSX)
