# Supplementary material for: Epigenetic and metabolic reprogramming of innate immune cells establishes immunological memory in the Schistosomiasis vector snail Biomphalaria glabrata
Source: PLoS Pathog. 2026 Aug 5;22(8):e1014471. doi: 10.1371/journal.ppat.1014471 (PMC13466059; doi:10.1371/journal.ppat.1014471)
Supplement: Supplementary file 1 [file ppat.1014471.s013.docx]

Review of MS PATHOGENS-D-00259

PLOS Pathogens

This is a great paper that digs into uncovering mechanisms of homologous immune priming /innate immune memory of *Biomphalaria glabrata* after exposure to *Schistosoma mansoni* with several coordinated methodologies. Figure 1 is highly appreciated—it diagrams the parasite development along with the experimental steps and really helps the reader understand the protocol. I highly support the publication of this work; however, have some recommendations for improvement.

Overall, while I found the manuscript to have reviewed immune priming/innate immune memory in other invertebrates quite well, they did not explain the fascinating details of this phenomenon in *Biomphalaria glabrata*-*Schsitosoma mansoni* especially outside of the specific model that was used. There are missing citations of work in the 1970s of the immune priming of *B glabrata* with schistosomes and other trematodes that should not be ignored. Also, explaining that the innate immune memory develops concurrently with the migration of the daughter sporocysts to the digestive gland.

There is a big picture item that is troubling regarding this concept with immune memory that the authors should address. It seems odd that immune memory would develop in snails that are 15 days into a chronic infection to prevent a new infection by the same parasite, but the response does nothing to quell the current infection, which is not even yet in patency and will rage on throughout the life of the snail. What would be adaptive of such a response? Earlier literature had not ruled out that this response could be orchestrated by the schistosome, but others have argued it is not adaptive for the schistosome. I am not convinced by these arguments. While there is clearly not an answer to this question, it is very critical to mention in the paper.

Similarly, regarding metabolic reprograming—while it does make sense that reprogramming to increase rapid energy could enable the immune response, it could also enable rapid parasite development. There are many examples of bacterial pathogens hijacking metabolism to increase their growth. Could the same be happening here?

In a couple places the author state that the acquired immunity is lifelong for the snail. I looked at the reference that was cited (#15). Unfortunately, that reference only used an experimental time course of 15 days, so “lifelong” data are not contained in the referenced paper. Ref 15 cited another paper that mentioned lifelong immunity—Sire et al 1998 Parasitology 117:117-122. However, this reference only tested the effect up to 8 weeks, which is clearly not the life of the snail. Is there a different citation for life long-immunity? Or perhaps just rephrase this to “long-lived immunity”

I also find that the paper lacks clearly stated hypotheses. While these are not strictly necessary, I believe they would help the reader understand the logic of combining all these technologies. In many sections, it seems the authors have underlying hypotheses that are simply not stated. Perhaps this would be helpful for the reader if they were stated.

There are several grammatical/awkward wording issues that need to be addressed. I have listed many in my review. The most critical is wording surrounding the sampling scheme. I found that in the way it was presented, I could interpret in multiple ways. Thus, this really needs to be addressed so that sampling is crystal clear.

**Methods**

Table 1 is confusing. For instance, the # of naïve snails (# of replicates)—one could interpret the hemocyte counts to be performed on one individual snail 37 times. I am assuming this simply just means that the samples were collected from individuals and 37 individuals. Maybe there is a different way to designate whether pools or individuals were used.

For the RNA seq-I am not sure I completely understand the comparisons of the analyses. So, first each was compared to a control. That makes sense. The secondary challenged snails were then compared to the 16-day primary infected snails. This also makes sense to remove the background of the first infection. However, if one wanted to compare the primary and secondary challenge, wouldn’t a direct comparison of these timepoints be necessary? This is definitely a complicated structure.

Line 718-721. This sentence is difficult to understand. I think is should stat that infection status was checked (not prevalence)—or that prevalence was measured?

Line 831. Still really confusing on the sampling. The biological replicate is the pool of 150 snails and a total of 3 mL was used? Please clarify.

**Results**

Line 175 Should state what the comparison is.

Figure 2 caption should state what the codes used are for the categories.

**Discussion:**

It would be highly beneficial if the authors discussed how their results differ/compare to other B. glabrata studies. For instance, there are a number of transcriptional studies of B. glabrata models. Are the results similar? Not details necessarily, but general patterns. Time curse of up/down regulation, the same type of genes up/down regulated.

Lines 546-550. This paragraph is very confusing, and I don’t understand what it is trying to say. The first sentence contains a bunch of clauses. The second sentence –is this talking about the current study??? Please restate this.

Line 551. “This observation”. What observation do you mean?

Line 567. What does partial to no mean?

Line 568. What does similarly refer to?

Line 580. What do you mean by epigenetic and metabolic changes coexisting? DO you mean that both occur in vertebrates, or do you mean they occur simultaneously or are correlated? Clarify.

Lines 590 on. Could metabolic changes be driven by parasitic infection? There is a lot of precedence for this in bacterial systems.

Lines 648 on. This paragraph links the metabolic changes with that of methylation change—however the methylation results are not super convincing. IS this over-reaching?

Lines 675-678. This is a strong assertion given the evidence presented.

Lines 669. The authors claim to have identified central molecular pathways that are indispensable for the induction of innate immune memory. I do not see how they have actually done this. They have found pathways correlated with innate immune memory. Please tone this down.

**Grammar:**

There are many grammatical and punctuation errors throughout the paper. I have listed many below. The authors should watch for use of plural v. singular as well as use of tense. There is mixing between tenses in the narrative.

Throughout the paper “infection kinetics” would be better than “kinetic of infection”. When “kinetic” is used as a noun, it is typically used in the plural as “kinetics”. In the paper some alternatives might be considered like time point or time course.

Also, for secondary challenge, the use of “challenge” is not adequate. Even the primary infection could be considered an infection “challenge”. Thus, it would be less confusing to the reader to specifically state “secondary challenge”.

Line 67. “evidences” should be “evidence”

Lines 71-72. “primed in a sustainable manner” is a confusing phrase. I recommend removing “sustainable manner”. Then explain what this phrase was supposed to mean like perhaps: *immune memory peaked around 14 days post infection and was sustained for at least 8 -weeks*.

This is seemingly small, but this change would enhance readability greatly—the use of “P” for primary infection works well, but the use of “C” for the secondary infection does not. “C” is so often linked with “control” and so I had to flip back and forth a lot to reconcile this difference. And I’m finally realizing after many hours of review that C must have been chosen for challenge. However challenge could also be used to describe the primary challenge so not ideal.

Line 149 “step” should be “steps”

Line 127 “increase” should be “increased”

Line 129, The use of “Only” is confusing—is this the “only” time point where the effect occurs, or are the authors referring to 24 hours as a short amount of time?

Line 132. Were should be was. (or increase to increases)

Line 141, Awkward construction. Perhaps just “there were no significant differences in counts of the 4 hemocyte types across time points.”

Line 174. I think it should be “differentially regulated” not just regulated.

Line 177 “kinetic” should be “time”

Line 208. Should this read is “still activated” rather than just “activated”. The former suggests genes that are activated during the primary response and remains upregulated during the secondary challenge. The latter would indicated that the sustained response is activated at the two week mark (so not during primary infection)

Line 513 hemolymphe should be hemolymph

Line 514 “infection s” should be “infections”

Line 535 “works” should be work

Line 570 Linked rater than link

Line 576 “were” should be “Are” and in line 577.

Line 628 Infection s” to “infections”

Line 652. “reversal of ATP production” should be “reduction of ATP production”

Line 672 occurs should be occur

Line 748. Duplicate, not duplicates. Does this sentence mean that there were duplicate pools or that the snails of one pool were bled twice?

Line 793. When should be while

Line 794. Secondary should be secondarily.

Line 803. Perhaps “Pellets of nuclei were immediately submitted…”
