# Supplementary material for: Epigenetic and metabolic reprogramming of innate immune cells establishes immunological memory in the Schistosomiasis vector snail Biomphalaria glabrata
Source: PLoS Pathog. 2026 Aug 5;22(8):e1014471. doi: 10.1371/journal.ppat.1014471 (PMC13466059; doi:10.1371/journal.ppat.1014471)
Supplement: Supplementary file 2 [file ppat.1014471.s015.docx]

**Response to Reviewers**

Paper # PPATHOGENS-D-26-00259.

Point by point rebuttal to the editor and reviewer comments.

**Paper title:**

Epigenetic and metabolic reprogramming of innate immune cells establishes immunological memory in the Schistosomiasis vector snail *Biomphalaria glabrata*.

**Additional Editor Comments:**

Expert reviewers have evaluated your manuscript and consider that your study provides some very interesting clues regarding the functional aspects of immune priming in Biomphalaria snails. However, they also identified some weaknesses in the manuscript that need to be addressed.

For example, reviewers suggested that you:

- Edit the introduction to clarify the study hypotheses and better reflect the current state of the field.

- Include additional literature relevant to the field.

- Revise the discussion to include additional interpretations of the results and use a more cautious tone.

- Revise the number of figures to ensure that they are relevant for conveying the main results described in the manuscript.

**All the requested modification shave been carefully addressed. We hope that the modifications described in the following rebuttal will be considered as satisfactory for you and the reviewers.**

**Journal Requirements:**

1) Please provide an Author Summary. This should appear in your manuscript between the Abstract (if applicable) and the Introduction, and should be 150-200 words long. The aim should be to make your findings accessible to a wide audience that includes both scientists and non-scientists. Sample summaries can be found on our website under Submission. Guidelines:

<https://journals.plos.org/plospathogens/s/submission-guidelines#loc-parts-of-a-submission>

**DONE**

2) We do not publish any copyright or trademark symbols that usually accompany proprietary names, eg ©, ®, or TM (e.g. next to drug or reagent names). Therefore please remove all instances of trademark/copyright symbols throughout the text, including: - ® on pages: 21, and 22.

**DONE**

3) Please upload all main figures as separate Figure files in .tif or .eps format. For more information about how to convert and format your figure files please see our guidelines: <https://journals.plos.org/plospathogens/s/figures> : **DONE**

4) Some material included in your submission may be copyrighted. According to PLOS's copyright policy, authors who use figures or other material (e.g., graphics, clipart, maps) from another author or copyright holder must demonstrate or obtain permission to publish this material under the Creative Commons Attribution 4.0 International (CC BY 4.0) License used by PLOS journals. : **DONE**

Potential Copyright Issues:

i) Please confirm (a) that you are the photographer of 1, or (b) provide written permission from the photographer to publish the photo(s) under our CC BY 4.0 license.

**All photographs of the mollusc were taken by the first author of this article. All figures and tables were either created by the authors or reproduced under a Creative Commons license (see acknowledgment).**

ii) Figures 1, 4, and 12. Please confirm whether you drew the images / clip-art within the figure panels by hand. If you did not draw the images, please provide (a) a link to the source of the images or icons and their license / terms of use; or (b) written permission from the copyright holder to publish the images or icons under our CC BY 4.0 license. Alternatively, you may replace the images with open source alternatives. See these open source resources you may use to replace images / clip-art:

- https://commons.wikimedia.org

- <https://openclipart.org/>.

**The figures cited have been revised to comply with the recommendations. Figure 12 has been removed. All photographs now included in Figures 1 and 4 were taken by the authors in the laboratory. The figures, drawings, and graphical elements were either created by the authors or adapted from resources distributed under a Creative Commons license.**

**To ensure proper attribution, we have added the following acknowledgment to the manuscript:**

**“Image adapted from Servier Medical Art (**[**https://smart.servier.com/**](https://smart.servier.com/)**), licensed under CC BY 4.0 (**[**https://creativecommons.org/licenses/by/4.0/).”**](https://creativecommons.org/licenses/by/4.0/).%E2%80%9D)

5) Thank you for stating that "Data are accessible on the repository SRA (sequence read archive) from NCBI under the BioProject ID PRJNA1422672." Please note that, though access restrictions are acceptable now, your entire data will need to be made freely accessible if your manuscript is accepted for publication. This policy applies to all data except where public deposition would breach compliance with the protocol approved by your research ethics board. If you are unable to adhere to our open data policy, please kindly revise your statement to explain your reasoning and we will seek the editor's input on an exemption. Please be assured that, once you have provided your new statement, the assessment of your exemption will not hold up the peer review process.

**Ok for making the data freely accessible when the paper will be published.**

6) Please amend your detailed Financial Disclosure statement. This is published with the article. It must therefore be completed in full sentences and contain the exact wording you wish to be published.

**Done**

1) State what role the funders took in the study. If the funders had no role in your study, please state: "The funders had no role in study design, data collection and analysis, decision to publish, or preparation of the manuscript."

**Done**

7) Please ensure that the funders and grant numbers match between the Financial Disclosure field and the Funding Information tab in your submission form. Note that the funders must be provided in the same order in both places as well.

**OK**

**Reviewers' Comments:**

**Comments to the Authors:**

**Please note that one review is uploaded as an attachment.**

Reviewer's Responses to Questions

**Part I - Summary**

Please use this section to discuss strengths/weaknesses of study, novelty/significance, general execution and scholarship.

**Reviewer #1**: Memory within innate immune systems is an important phenomenon. It comes as 'trained immunity' in vertebrates and 'immune priming' in invertebrates. The latter consists of a large variety of mechanisms in the different invertebrate taxa. In most cases where phenomena of immune priming have been shown, the underlying mechanisms are not known in details. The present study thus provides an important step towards such better mechanistic

knowledge. It specifically asks whether mechanisms that are underlying vertebrate trained immunity might also be present during some phenomena of invertebrates immune priming. The authors make use of a powerful system for studying immune priming, namely the snail Biomphalaria glabrata and its trematode parasite Schistosoma mansoni. Using a combination of hemocyte RNA sequencing, bisulfite sequencing, ATAC sequencing and metabolomics,

they indeed find strong indications that metabolic and epigenetic reprogramming of hemocytes seem to occur, pointing towards similarities with vertebrate trained innate immune cells.

Given the almost complete lack of previous evidence for such similarities, these results are novel and highly significant. Another strength of the study is the model system, because it consists of a natural host-parasite system. However, this is also bound to a certain (although minor) weakness, because priming is induced by infection, and it is not possible to disentangle infection effects from priming that might occur through non-infectious agents. The experiments are generally well-executed, with one larger limitation: there doesn't seem to be non-infected controls taken at the same time points as the samples that were taken after priming and challenge. Rather, it seems that all comparisons were done with naive snails from the first time point. At least this is what seems shown in Fig. 1. Such a design would not control for potential changes over time that could have occurred in naive snails as well. The results are presented and discussed in an appropriate manner, but I do have a minor concerns regarding the introduction and the somewhat limited focus of the discussion that doesn't sufficiently account for alternative explanations. Overall, this is a novel and highly significant study that is well-suited for the journal PLoS Pathogens.

**Many thanks for your comments on the quality of the experiments conducted in the present study. It was a very hard and long work conducted over the last 5 years. Concerning the point raised about the controls : there is two important controls used in this study, (i) non-infected controls that were used to compare with the infected snails over the 15 days of the experiment and (ii) the primary infected snails at 16 days used as control to compared with changes in secondary challenged snails at 6h, 12h and 24h after the challenge.**

**Non-infected snails were recovered from the breeding tanks at the same time than the experimental infected snail groups, and were used for hemocyte recovery and frozen in liquid nitrogen at the same time than the 24h infected time point, but indeed we do not recovered non-infected control snails for each time point of the infection kinetic, this would represent a too high number of snails used for the experiment. Moreover, knowing that snails live around 2 years in our laboratory conditions we hope that the experiment done over 16 days would not be associated with strong modifications of their metabolism, transcription, of epigenetic statute or even less than what could be induced by or associated to the infection.**

Reviewer #2: See attachment

**All the comments of reviewer #2 will be addressed at the end of this rebuttal.**

Reviewer #3: I will begin my review by pointing out that I do not have personal expertise with many of the methods used in this paper, although I understand them. So, I am taking their experimental results at face value. If there are methodological flaws, I defer to the opinion of other reviewers on that. I can comment on the overall experimental design and the importance of the results. I thought the experimental design (Fig. 1) was nice in that it allowed them to define when in the process of first and second challenges, over 16 days, the major changes in gene expression and epigenetic modifications take place. Combining RNAseq, ATAC-seq, WGBS and the targeted metabolomics allowed them to take a comprehensive look at the changes involved in the shift to a humoral response seen in IIM. They found substantial epigenetic and metabolomic changes after the first infection, but not the second. After the second challenge there is a massive upregulation in expression of many genes. All the methods point to shifts in cell metabolism, similar to what is observed in vertebrate trained immunity, as key processes in snail IIM. What type of epigenetic change reprogrammed those cells to respond that way still needs to be explored. As is typical, DNA methylation changes didn’t correlate with gene expression changes. However, the ATAC-seq results were quite interesting. Opening sites overlapped mostly with genes upregulated in their “sustained response” profile, and less so with the “immune shift” response, which I don’t think was expected. There are still many questions to be answered about epigenetic control, but this study was a nice start. The author speculate quite a bit in the Discussion. I have no problem with that, as I think that is the appropriate place to lay out new ideas and to propose hypotheses to test in the future. However, to avoid criticism, they might want to use more circumspect language, couching anything that isn’t a concrete result with phrases such as “It appears that …”, or “We speculate that…” or “We hypothesize that…”. This paper appears to be the first on molluscs that investigates how IIM is achieved, and is one in what is still a small literature on IIM in invertebrates. That their results point to metabolic shifts similar to what is seen in vertebrate trained immunity was particularly interesting. What they found should be of interest to researchers studying a variety of organisms. Overall, I enjoyed this thought-provoking paper, and don’t see much to criticize. One small comment is that there are a lot of figures in the manuscript. Some of the figures come across as just pretty pictures, rather than adding substantially to understanding of the results. For example, they might want to explain what is special about the genomic regions displayed in Fig. 5A and 7A & B. Using the figure legends to tell the reader what the reader should take away from each figure can be useful.

**Many thanks for your clever reading of the paper and your perfect understanding of the central results of this study. You are right that the discussion is speculative and we follow your recommendations to be more cautious. The discussion was thus modified in that way.**

**Concerning the figures we agree with your comments that some figures were just to illustrate how were conducted the experiments but not linked directly with the results. Thus, figures were reduced and more substantially dedicated to the results.**

**Part II – Major Issues: Key Experiments Required for Acceptance**

Please use this section to detail the key new experiments or modifications of existing experiments that should be absolutely required to validate study conclusions. Generally, there should be no more than 3 such required experiments or major modifications for a "Major Revision" recommendation. If more than 3 experiments are necessary to validate the study conclusions, then you are encouraged to recommend "Reject".

**Reviewer #1**: I don't see any new experiments that would be absolutely required to validate the study conclusions. Of course, we could always ask for more, such as functional validation of some key mechanisms. But this would be too much to ask for, given the complexity of the host studied here.

**Thanks.**

**Reviewer #2**: See attachment

**All the comments of reviewer #2 will be addressed at the end of this rebuttal.**

**Reviewer #3**: none

**Part III – Minor Issues: Editorial and Data Presentation Modifications**

Please use this section for editorial suggestions as well as relatively minor modifications of existing data that would enhance clarity.

**Reviewer #1:** I have a few points that could be addressed with minor modifications of the introduction, the presentation of the experimental design and data, as well as the discussion. All of these points can be addressed with relatively minor modifications of the manuscripts and don't need additional experimentation.

(1) I personally don't like how the diversity of immune priming phenomena is presented as a limitation. Phrasing such diversity as a problem creates unnecessary controversy. Diversity of mechanisms underlying immune priming rather is a biological fact. There are many different mechanisms involved (in the many different taxa), so the call for universality (of model organisms studied) and the illusion that there might be a uniform mechanistic underpinning is doing harm to the field and hinders the power to gain insight into the many different mechanisms. I agree that some immunologists share the scepticism presented in the introduction (this may be mentioned) but in my view, it is not a suitable introduction (and justification of this study). Why not phrasing the open question to what extent trained immunity - like processes are involved also in invertebrate priming? This provides sufficient novelty and doesn't give the wrong impression that all invertebrate immune priming is the same and based on trained immunity - like processes. I know that the authors are well aware of these aspects, so why not also introducing the topic that way?

**We are agreeing that the introduction is narrowed to trained immunity and that this could indeed be a problem to reduce immune priming to a limited set of potential mechanisms. We tried to follow your recommendations to open-up the question and thus we modify the introduction accordingly (see lines 107-126).**

(2) Related to this, the introduction plays down the increasing knowledge of mechanistic, molecular underpinnings of immune priming in more and more systems. These mechanisms are often taxon-specific and depend on the type of pathogens, route of infection, etc. Why not doing justice to these studies and naming at least some examples? This doesn't decrease the relevance of the question addressed here, namely whether mechanisms similar to trained immunity might also be relevant for invertebrates, which remains to be an exciting and novel Question.

**This was also modified accordingly to the point (1) (see lines 107-126).**

(3) The potential limitation in study design mentioned above (no control samples taken at the exact same time point after priming and challenge) should be addressed more clearly. First, it has to become clear from the results and methods description whether this was indeed the case? If so, it should be discussed why this is not regarded a limitation (I assume the authors have arguments, otherwise they would have taken these time-matched controls). Related to this, it doesn't become clear whether any kind of 'sham infection' was done as control?

**As explained above indeed controls were not taken for each time points of the kinetic, the main argument being the snail’s lifespan related to the 16 days of the experiment, more over the naive samples were taken from the breeding tanks, hemolymph recovered and frozen in liquid nitrogen at the time point corresponding to 24h of the kinetic of infection, this being also important for comparing to the short kinectic time points (6h, 12h, and 24h) and also to the longer ones (4 days and 15 days). Same procedure was done for the control used for the challenge, we recovered also in the same manner a control at 16 days after primary infection to compare with the challenge at 6h, 12h and 24h (see lines 897-898).**

**For the shame-infection, sorry but we are not sure of what you expect here. The infections were natural; there is no human intervention in such infection procedure. The miracidium stages of the parasite are put in contact with the snail and let for 3h, time necessary for being sure that the parasite penetrate throw the snail tissue and effectively infect the snail. Thus, it is impossible to conduct a shame infection. The only point is that the naive snails are submitted to the same procedure without putting parasites (This is now more clearly stated in the material and methods section) (lines 884-887).**

(4) Please provide more details regarding the infection success. Where all exposed snails infected (when primary infected)? From line 708 it seems not? If that is the case, what happened to exposed-but not infected snails? Where they not studied any further? If not, what are the arguments why not? If many snails were exposed-but not infected, this could bias the subgroup of infected snails and effects might be partially due to this selection effect. I assume the authors have thought about this and have good arguments why this doesn't pose any Problems.

**Thank you for this comment. Indeed, it was extremely important that all the snails due to be exposed to the parasite a second time had been infected since the first infection.**

**To ensure this, we rely on two checks. The first involves testing the prevalence and intensity of each infection using a group of control snails selected at random from the experimental snail group (lines 899-900). In all experiments, the infection prevalence was at 100% following primary infection. The second check is carried out visually, as noted in lines 899-900, by looking through the shell to confirm the presence of the parasite.**

(5) It should be acknowledged that proof for epigenetic reprogramming is currently still a bit limited in the system studied here. The role of CpG methylation is unclear (methylation rates are low and unrelated to gene expression). ATAC seq provides valuable information about chromatin accessibility but it doesn't provide the mechanisms causing these differences, such as histone modifications. I am not asking for futher experiments looking into this, because it would be challenging in this system. But these limitations should be spelled out even more clearly. In Fig. 12 epigen. modification are shown that were not studied here (I see that this is described as 'theoretical' but still, why not sticking to what was studied here?)

**We are totally agreeing with this comment, and clearly the role of DNA methylation in invertebrate is a real actual challenging question about their potential role in regulation of gene expression. For ATACseq the function is more classically related to expression, but how this opening and closing of the chromatin is regulated is a complicated question. To state about these limitations, we add some comments in the discussion section (lines 683-713).**

**For the figure 12, we indeed want to use it as a description of the hypothetical processes that may occur in our system, as perspectives and open questions for what is needed now in Biomphalaria snail to go further in this question of molecular support of immune priming. But we agree that the paper is not a review or an opinion paper and thus we decide to follow your advice and we removed the figure 12.**

(6) The discussion is very much focused on trying to proof epigenetic and metabolic reprogramming, which is fair enough, but I would prefer a slightly more open approach. For example, how is the potential involvement of these processes interpreted in the light of previous findings in this system (FREPs etc.?). What about the (lack of) specificity, e.g. for schistosome strains?

**We added some discussion about the role of immune complex constituted by FREP / TEP and Biomphalysin in the recognition and the killing of S. mansoni sporocysts. Interesstingly those molecules were overexpressed early after the secondary challenge and could thus participate in the efficient recognition and killing of the parasite (humoral pathway) (see Lines 660-675).**

(7) The absence of changes in hemocyte types depends on the reliabilty to discriminate hemocytes. It is quite likely, that improved techniques (such as scRNAseq) would differentiate hemocytes that are morphologically indistinguishable (as has been shown in other invertebrates). This is important, because the techniques chosen here describe bulk changes (in the mixed population of hemocytes). It thus remains unstudied here whether observed changes are due to changes (of expression, of open chromatin, etc) occurring to individual cells, or whether cells with certain expression patterns might become more numerous after priming (while expression of individual cells itself might remain unchanged). This distinction is relevant regarding the interpretation of the results (such as similiarity with trained immunity) and should at least be discussed in more detail.

**Thanks for this comment indeed we have done an import work on hemocytes using single cell transcriptmic recently. Your comment help to put in perspective such work with the bulk RNAseq done in the present paper. Thus, we add some elements of discussion concerning this strong transcriptomic plasticity compared to the morphological diversity that was demonstrated in *B. glabrata* snail hemocytes. Please see lines 681-694**

(8) ATAC sec compared P24h and C24h vs naive while methylation compares P16d vs naive. Why were different timepoints chosen? I guess there are good reasons, please mention them.

**Based on previous unpublished data and preliminary results done in our lab on changes in chromatin structure we observed that such structural changes occurred very quickly after infection. Thus, for the ATAC-seq we decide to see the changes after solely 24h of infection following the primary infection and following the secondary challenge. Such timing appeared as particularly relevant as we can see huge changes following primary infection and very limited changes following the challenge indicating that the first stress of infection induced strong modifications of chromatin structure in hemocytes that remained stable for days even following parasite secondary challenge. For DNA methylation this is totally different. First the WGBS approach necessitates a huge number of snail hemocytes to produce the required DNA amount for the analysis, we were not able to analyse more kinectic points that the ones investigated in the present paper. Moreover, changes in methylation is a process that could take time to occurs, and in our model the level of DNA methylation is low (doi: 10.1186/1756-3305-6-167.), in addition, we were expecting that changes in cell metabolism may induce changes in epigenetic marks as observed in Trained Immunity and this would takes time to occurs. Thus, to optimize the chances to see a significant modification of the DNA methylation, we decide to compare naive snails to P16d primary infected snails. Finally, this WGBS approach is also very costly, and this was a second limiting step for us, in selecting time points to analyse.**

(9) There are some further minor points:

Title: either put a comma after 'glabrata' or write '…in the schistosomiasis vector snail Biomphalaria glabrata'.

**Done**

L. 128: reduce number of digits : **Corrected accordingly**

L. 146 ff and L. 192 ff. Fig 1 and 3: improve this figure legends, partially awkward style

**The legends are rephrased as follows and Figure 2 and 3 are now merged into Figure 2.**

**Figure 1. Experimental protocol:**

**Infection kinetics used to characterize the innate immune memory response of the snail *Biomphalaria glabrata* from brazil(BgBRE2) primary infected and secondary challenged with its sympatric *Schistosoma mansoni* parasite (SmBRE). The upper part of the figure illustrates parasite development following primary infection and upon secondary challenge, together with associated hemocyte responses. After primary infection, miracidia penetrate the snail tegument; a majority are rapidly eliminated by hemocyte encapsulation, while the remaining parasites develop into primary sporocysts (SpI), which give rise to secondary sporocysts (SpII). Mature SpII subsequently migrate to the digestive gland to produce cercariae. Upon secondary challenge, invading miracidia are rapidly recognized and eliminated, and no reinfection occurs (*11, 15, 26)*. The lower part of the figure shows hemocyte sampling time-points for counting, RNAseq, ATACseq, WGBS and metabolomic experiments were indicated along the kinetic of primary infection and secondary challenge using purple, pink, yellow, green, and blue rectangles respectively. Red flashes indicated the time point of primary and secondary infections using 10 miracidia of SmBRE.**

**Figure 2. Changes in circulating hemocyte abundance and transcriptional reprogramming during IIM acquisition**

**A.) Mean number of circulating hemocytes per µl of hemolymph (Cell/µl) in BgBRE2 snails following primary infection and secondary challenge by SmBRE parasites. Error bars correspond to standard deviation. N: Number of snails used as replicates. Different letters (a, b, c) indicate statistical differences (P < 0.05) estimated by Dunn post-hoc test and FDR benjamini-Hochberg correction. B.) Representative morphological profiles of B. glabrata hemocyte populations stained with MCDh, red scale bars correspond to 10 µm. Associated graphs show the mean percentage of each population according to infection kinetic time points (N=10 per time point). C.) Histogram showing the number of differentially expressed genes (DEGs) in infected snails (primary infected and secondary challenged) compared with naïve snails during infection. Numbers above bars indicate the total number of significant DEGs. Blue and yellow represent up- and downregulated genes, respectively. D.) Heatmap of significant DEGs in infected snails compared with naïve snails during infection. Colours indicate log2 fold change (log2FC), with blue and yellow representing up- and downregulation, respectively. Genes are grouped into five clusters, depending on transcriptomic response patterns during the kinetics of infection. E.) Histogram showing the number of significant DEGs in secondary challenged snails compared with primary infected snails (P16d) over time. Numbers above bars indicate total DEGs. Blue and yellow represent up- and downregulated genes, respectively. F.) Venn diagram showing shared enriched functions among upregulated genes across the three conditions. G.) Heatmap of significant DEGs in secondary challenged snails compared with primary infected snails (P16d) during reinfection with SmBRE. Colours represent log2FC. Genes are grouped into five clusters, including four clusters of upregulated genes and one cluster of downregulated genes.**

L. 166: Fig 2B: same N as in Fig2A?

**Legend corrected as follow:**

**(N=10 per time point)**

l. 276 and above: rationale for sorting genes into recalled, sustained and shift has to be presented in a clearer manner

**Text rephrases as follow:**

**To characterize transcriptional dynamics following secondary challenge, DEGs were classified into three categories based on their temporal expression profiles: recalled, sustained, and shift responses. Recalled genes show transient expression during primary infection and re-induction upon secondary challenge. Sustained genes are induced during primary infection and maintained at similar levels after the secondary challenge. Shift genes represent transcripts only expressed during secondary responses, reflecting a reprogramming of hemocyte functions. Following secondary challenge with S. mansoni, hemocytes of *B. glabrata* exhibited all three response types. The shift response was the most important, emerging as early as 6 h post-secondary challenge, peaking at 12 h, and declining by 24 h (Figure 2G). Functional annotation of these DEGs revealed significant changes between C6h and C12h, consistent with a cellular energy metabolism reorganization and a transition toward humoral immune responses (Figure 3).**

l. 290 what does 'mosaic type' mean - please explain

**Text rephrases as follow:**

Hemocyte DNA methylation is predominantly in a CpG region and displayed a mosaic pattern, characterized by alternating methylated and unmethylated genomic regions, as previously described in whole snails.

l. 318, interesting point, provide more details (maybe in the discussion): what is known about the function of methylation in snails?

**Paragraph starting line 568 rephrase as followed to provide more details in the discussion:**

**Concerning DNA methylation for non-vertebrates, the role and function of DNA methylation appeared as a complex black box. The detailed molecular mechanisms through which DNA methylation signaling contributes to regulate gene expression is yet to be explored. DNA methylation has been demonstrated as playing a role in protecting animal genomes against the harmful effects of transposon activity or to environmental stressors, but the link with transcriptional regulation remains weak or unclear (41-44). DNA methylation may be also implicated in genome stability and environmental responses. Experimental alteration of methylation patterns in *Biomphalaria glabrata* has been shown to affect phenotypic traits and gene function (Luviano et al., 2021) https://doi.org/10.1186/s13072-021-00422-7 . Moreover, components of the DNA methylation machinery are modulated during host–parasite interactions, suggesting a role in the response to *Schistosoma mansoni* infection (Geyer et al., 2017) https://doi.org/10.1371/journal.pntd.0005246. Consistent with this, we did not observe a clear correlation between differentially methylated genes and transcriptional changes in *Biomphalaria glabrata*. Only a limited proportion of differentially methylated genes are correlated with differentially expressed genes, with heterogeneous expression patterns. Nevertheless, differentially methylated genes identified in this study were associated with key signaling pathways involved in immune cell activation, suggesting that DNA methylation may contribute to the regulation of hemocyte functional states. Altogether, these findings support the idea that DNA methylation does not act as a direct driver of transcriptional changes but may instead participate in the priming or stabilization of immune responses, potentially contributing to innate immune memory. The molecular mechanisms underlying these effects, however, remain to be elucidated.**

l. 368 the direction of the described effects is unclear; I assume 'opening' means open in treatments (here C24h) compared to control (as it is written, it seems the other direction was meant?)

Yes, that’s exactly right. When we talk about an open region, we mean a region of chromatin that becomes more accessible compared to naive hemocytes.

**Text rephrases as followed for line 488:**

**This observation is strengthened by the fact that 94.3% of the genomic regions showing increased chromatin accessibility were associated with a predicted gene within these regions for P24h and C24h samples (Figure 7D).**

**And line 494:**

**We observed 2556 opening regions at P24h and 3091 opening regions at C24h, both compared to naive snail’s hemocytes.**

- l 386 which phenotype? transcriptome?

**Here we were referring to the phenotypic changes observed following challenge, i.e. a cellular response following the first infection and a humoral response following the second. The functions supported by the genes in these open regions of chromatin appear to be linked, on one hand, to the response to the parasite (immunity) and, on the other hand, to the reorganization of hemocytes and the regulation of gene transcription.**

**Text rephrase as followed, line 512: All these enriched functions fit perfectly with the phenotypic changes (i.e. a shift from a cellular response by hemocytes to a humoral response) that were expected to occur in hemocytes following primary infection and IIM acquisition.**

- l 472: color scheme in fig 10D?

**Corrected as followed:**

**D.) Schematic representation of major metabolites, related to glycolysis, TCA cycle and urea cycle, differentially represented in the P24h condition with a blue-to-red gradient representing the average quantity of each metabolite.**

- l. 682 specify in more detail what kind of 'further work'

**We thank the reviewer for this suggestion. We have clarified the nature of the future work required by specifying that comparative studies across a broader range of animal phyla and host–pathogen systems will be necessary to assess the conservation and evolutionary origin of the molecular mechanisms underlying innate immune memory.**

**Line 930: “Therefore it is now needed to obtain an overview of this new concept and understand the nature and origin of these conserved characteristics by analysing the molecular basis of innate immune memory in a growing number of phyla of the animal kingdom, taking into account the diversity of the interactions studied.”**

- Table 1 is unclear and confusing regarding the exact levels of replication, etc. Some Figures are truncated in pdf but ok in pptx

**All figures and tables will be uploaded separately in accordance with the publisher’s guidelines. It appears that the PDF version has been truncated.**

**Table 1 modified as follows:**

**N : number of replicates: correspond to the number of biological replicates done for each experiment; conditions: correspond to the time points analysed in the kinetic of primary infection and secondary challenge.**

Fig 3E how were enriched function ‘selected’?

**The functions highlighted in this figure were selected based on their significance in the enrichment analysis for each section of the heatmap. To reduce the number of function names and to clarify which major enriched functions are present, we used Revigo.**

Discussion/Literature:

There is a recent study that also indicates the involvement of epigenetic reprogramming that the authors may wish to cite: Deng Z, Luo J, Zhang Y, Li C, Deng S, He J, He Z, Wu W, Jiao R, Liu J. 2025. PGRP–SC–

mediated innate immune memory against pathogenic bacteria in Drosophila. ELife 1:3. DOI:

<https://doi.org/10.1016/j.hlife.2025.09.003>

**Thanks for this recent article which has been added to the present study.**

**Reviewer #2**: Lots of grammatical issues. See attachment.

**Reviewer #3:** see main comments

**#Reviewer 2**

Review of MS PATHOGENS-D-00259

PLOS Pathogens

This is a great paper that digs into uncovering mechanisms of homologous immune priming /innate immune memory of Biomphalaria glabrata after exposure to Schistosoma mansoni with several coordinated methodologies. Figure 1 is highly appreciated—it diagrams the parasite development along with the experimental steps and really helps the reader understand the protocol. I highly support the publication of this work; however, have some recommendations for improvement.

Overall, while I found the manuscript to have reviewed immune priming/innate immune memory in other invertebrates quite well, they did not explain the fascinating details of this phenomenon in Biomphalaria glabrata-Schistosoma mansoni especially outside of the specific model that was used. There are missing citations of work in the 1970s of the immune priming of B glabrata with schistosomes and other trematodes that should not be ignored. Also, explaining that the innate immune memory develops concurrently with the migration of the daughter sporocysts to the digestive gland.

**To address this point, we have revised the Introduction (lines 127) to include additional historical and biological context specific to this model : “Studies on the freshwater snail *Biomphalaria glabrata*, have provided some of the most fascinating examples for invertebrate IIM against the agent of human schistosomiasis (Gourbal et al., 2018, Pinaud et al., 2016). Early studies conducted in the 1970s already reported that *B. glabrata* snails infected with trematodes like *Echinostoma* parasites, develop resistance to subsequent infections, a phenomenon referred to as “acquired resistance” (Lie et al., 1982 https://doi.org/10.2307/3280906). It was only several years later that this phenomenon was demonstrated against schistosoma mansoni (Sire et al., 1998, DOI:** [**10.1017/s0031182098002923**](https://doi.org/10.1017/s0031182098002923)**). Although the mechanisms underlying this resistance were unknown at the time, these observations strongly look like what is now described as innate immune priming. Indeed, snails that have previously been exposed to the parasite *Schistosoma mansoni*, showed increased resistance upon secondary exposure (Pinaud et al., 2016, Portela et al., 2013). This IIM response is acquired in a time dependent manner, persists throughout the snail’s life and is supported by a change from an immune cellular encapsulation response after primary infection toward a hemocyte-mediated humoral immune response after a secondary challenge (Pinaud et al., 2016). Interestingly, the establishment of this immune memory coincides with parasite development, particularly the migration of daughter sporocysts to the digestive gland, suggesting a close link between parasite progression and long-term modulation of host immune responses. However, earlier studies have also shown that exposure to primary sporocyst (SpI) extracts is sufficient to induce a protective response (Pinaud et al., 2019,** [**https://doi.org/10.1016/j.dci.2018.12.001**](https://doi.org/10.1016/j.dci.2018.12.001)**), indicating that early parasite-derived signals can initiate immune priming.”**

There is a big picture item that is troubling regarding this concept with immune memory that the authors should address. It seems odd that immune memory would develop in snails that are 15 days into a chronic infection to prevent a new infection by the same parasite, but the response does nothing to quell the current infection, which is not even yet in patency and will rage on throughout the life of the snail. What would be adaptive of such a response? Earlier literature had not ruled out that this response could be orchestrated by the schistosome, but others have argued it is not adaptive for the schistosome. I am not convinced by these arguments. While there is clearly not an answer to this question, it is very critical to mention in the paper.

**In our experimental design, the secondary challenge was performed at 15 days post-primary infection to ensure that immune memory was fully established and to avoid confounding effects of the initial immune response. However, previous studies have shown that resistance to reinfection start around 7 days post-infection (Sire et al., 1998), suggesting that the mechanisms underlying immune priming are initiated early during infection. One possible explanation is that the parasite undergoes developmental and molecular changes within the host that allow it to evade or tolerate the host immune response, as previously described ( doi: 10.1016/j.fsi.2015.01.036). In this context, parasite first infecting the host induce the activation of an efficient immune priming, that will be able to kill the parasite of the secondary challenge while being ineffective on the first entered parasite. This immune process is named concomitant immunity and especially well described in the interaction between *S. mansoni* adult worms and vertebrate hosts, the parasites can persist for many years in the immunocompetent host (https://doi.org/10.1038/nri843).**

Similarly, regarding metabolic reprograming—while it does make sense that reprogramming to increase rapid energy could enable the immune response, it could also enable rapid parasite development. There are many examples of bacterial pathogens hijacking metabolism to increase their growth. Could the same be happening here?

**We thank the reviewer for this comment. We agree that metabolic reprogramming could, in some systems, benefit the pathogen as well as the host, particularly when intracellular pathogens are able to directly manipulate host cell metabolism to support their own growth. However, this is less likely in our study because the parasite is not intracellular and therefore does not directly exploit the metabolic machinery of host cells.**

**Importantly, our analyses focus on metabolic changes occurring within hemocytes. In these immune cells, increased energy production is widely recognized as a hallmark of immune activation and has been described in the context of trained immunity in mammals. For this reason, we interpret the observed metabolic reprogramming primarily as a host-driven response that supports immune functions.**

**That said, we agree that infection can impose energetic costs at the whole-organism level and may lead to trade-offs in resource allocation. While such effects cannot be excluded, the cellular changes observed here are more consistent with immune activation than with direct parasite exploitation of host cell metabolism.**

In a couple places the author state that the acquired immunity is lifelong for the snail. I looked at the reference that was cited (#15). Unfortunately, that reference only used an experimental time course of 15 days, so “lifelong” data are not contained in the referenced paper. Ref 15 cited another paper that mentioned lifelong immunity—Sire et al 1998 Parasitology 117:117-122. However, this reference only tested the effect up to 8 weeks, which is clearly not the life of the snail. Is there a different citation for life long-immunity? Or perhaps just rephrase this to “long-lived immunity”

**We thank the reviewer for this comment. We agree that the references originally cited do not directly demonstrate protection throughout the entire lifespan of the snail. We have therefore added a more appropriate reference showing that protection can persist for at least 140 days after primary infection. Importantly, infected snails generally die earlier than uninfected individuals because of parasite development within the host. As a result, the duration of protection can only be assessed over the lifespan of infected snails. The available evidence indicates that protection is maintained throughout the life of infected individuals, and we have therefore retained the term “lifelong immunity” while clarifying this point and adding the additional reference in the revised manuscript.**

**Line 136: “This IIM response is acquired in a time dependent manner, persists throughout the snail’s life (at least 140 days after primary infection) and is supported by a change from an immune cellular encapsulation response after primary infection toward a hemocyte-mediated humoral immune response after a secondary challenge (doi: 10.1371/journal.ppat.1005361.).”**

I also find that the paper lacks clearly stated hypotheses. While these are not strictly necessary, I believe they would help the reader understand the logic of combining all these technologies. In many sections, it seems the authors have underlying hypotheses that are simply not stated. Perhaps this would be helpful for the reader if they were stated.

**Thank you for your comment; we have clarified our assumptions concerning this integrative omics approach more clearly in the introduction section of the article; we hope that you will find this more appropriate and satisfactory. Please see lines 162-179.**

There are several grammatical/awkward wording issues that need to be addressed. I have listed many in my review. The most critical is wording surrounding the sampling scheme. I found that in the way it was presented, I could interpret in multiple ways. Thus, this really needs to be addressed so that sampling is crystal clear.

Methods

Table 1 is confusing. For instance, the # of naïve snails (# of replicates)—one could interpret the hemocyte counts to be performed on one individual snail 37 times. I am assuming this simply just means that the samples were collected from individuals and 37 individuals. Maybe there is a different way to designate whether pools or individuals were used.

**Table clarified. See comment from reviewer 1**

For the RNA seq-I am not sure I completely understand the comparisons of the analyses. So, first each was compared to a control. That makes sense. The secondary challenged snails were then compared to the 16-day primary infected snails. This also makes sense to remove the background of the first infection. However, if one wanted to compare the primary and secondary challenge, wouldn’t a direct comparison of these timepoints be necessary? This is definitely a complicated structure.

**Thank you for this helpful comment. We agree that a direct comparison between primary- and secondary-challenged snails could be informative. However, such an approach would mix the effect of the challenge with the transcriptional changes already ongoing at P16d following the primary infection. For this reason, we used the P16d time point as a reference to better isolate the transcriptional response specifically associated with the secondary challenge.**

**In our RNA-seq analysis, comparing challenged snails to naïve snails allowed us to capture the overall transcriptional response across the infection kinetics. In parallel, comparing secondary-challenged snails to primary-infected snails at P16d enabled us to control for the transcriptional background induced by the developing parasite and to more precisely identify changes associated with the secondary challenge and innate immune memory.**

Line 718-721. This sentence is difficult to understand. I think is should stat that infection status was checked (not prevalence)—or that prevalence was measured?

**Yes, the infection prevalence (the number of infected snails divided by the number of exposed snails) was measured for each experiment. The data are not presented here because we verified that, in each experiment, the infections had worked normally, with a prevalence of 100% following exposure to 10 miracidia.**

Corrected as followed for clarity:

Line 972: the status of the infection was assessed by measuring its prevalence

Line 831. Still really confusing on the sampling. The biological replicate is the pool of 150 snails and a total of 3 mL was used? Please clarify.

**Yes, that’s right. The sentence has been modified as follows for greater clarity.**

**Line 1091: 3ml of hemolymph from three pool of 150 snails (biological replicates) were used for DNA extraction for each treatment.**

Results

Line 175 Should state what the comparison is.

**The sentence has been modified as follows for greater clarity.**

**"Briefly, 207, 308, 108 and 6 genes were up-regulated at P6h, P12h, P24h and P96h respectively, compared to the naive control.”**

Figure 2 caption should state what the codes used are for the categories.

**In the revised manuscript, the color coding originally used in Figure 2 has been removed to improve clarity and readability. As a result, the categories are now directly identified within the figure, and no color-coded legend is required.**

Discussion:

It would be highly beneficial if the authors discussed how their results differ/compare to other B. glabrata studies. For instance, there are a number of transcriptional studies of B. glabrata models. Are the results similar? Not details necessarily, but general patterns. Time curse of up/down regulation, the same type of genes up/down regulated.

**We thank the reviewer for this valuable suggestion. We have expanded the Discussion section to better place our findings within the context of the existing literature on *Biomphalaria glabrata*–*Schistosoma mansoni* interactions. In particular, we now discuss the expression of key immune effectors, including the FREP/TEP/Biomphalysin complex, which has previously been implicated in parasite recognition and killing. We also highlight that these molecules were overexpressed early after the secondary challenge, consistent with an enhanced immune response (see Lines 660–675).**

Lines 546-550. This paragraph is very confusing, and I don’t understand what it is trying to say. The first sentence contains a bunch of clauses. The second sentence –is this talking about the current study??? Please restate this.

**The sentence has been modified as follows for greater clarity.“To our knowledge, most studies investigating epigenetic regulation in invertebrates following infection have focused on the expression of epigenetic enzymes or pathways, rather than on direct measurements of epigenetic marks. As a result, evidence for the involvement of epigenetic modifications in immune priming remains indirect, and the underlying mechanisms are still poorly understood (Castro-Vargas et al., 2017, Contreras-Garduno et al., 2016).”**

Line 551. “This observation”. What observation do you mean?

‘**This observation’ refers to the previous paragraph, which highlights the lack of data in the literature on the links between epigenetic modification and immune priming. The sentence has been modified as follows for clarity.**

**Modified sentence: Given this lack of direct evidence linking epigenetic modifications to immune priming, we investigate the impact of epigenetic pathways through analysing differentially methylated regions and chromatin accessibility between naïve, primary infected and challenged snails.**

Line 567. What does partial to no mean?

**The sentence has been modified as follows for clarity.**

**“but the link with transcriptional regulation remains weak or unclear”**

Line 568. What does similarly refer to?

**We wanted to show that, as with other organisms in which methylation has been studied, no studies have demonstrated a correlation between methylation in Biomphalaria and gene expression.**

**The paragraph has been rewritten (see Reviewer 1’s comment).**

Line 580. What do you mean by epigenetic and metabolic changes coexisting? DO you mean that both occur in vertebrates, or do you mean they occur simultaneously or are correlated? Clarify.

T**he sentence was intended to say that epigenetic and metabolic changes are functionally interconnected in vertebrates.**

**The word ‘coexist’ has been replaced by ‘tightly linked’**

Lines 590 on. Could metabolic changes be driven by parasitic infection? There is a lot of precedence for this in bacterial systems.

**We agree that metabolic changes can be driven by parasitic or microbial infections, as widely described in bacterial systems where pathogens can hijack host metabolism to promote their own growth.**

**In our system, however, several observations suggest that the metabolic changes we report are more likely associated with host immune activation. First, these changes occur rapidly following secondary challenge, within a timeframe that is more consistent with immune activation than with parasite development. Second, secondary infections do not lead to successful parasite establishment, suggesting that the observed metabolic shifts are unlikely to benefit the invading parasite. Finally, these changes appear to be largely restricted to hemocytes, making them less accessible to the parasite, and functional annotation of differentially expressed genes indicates enrichment in pathways typically associated with immune responses and cellular activation.**

**Nevertheless, we cannot exclude that some aspects of host metabolic reprogramming may be due to (i) a collateral effect (presence of parasite or overall pathological effect), (ii) manipulated by the parasite for his own profit, or (iii) link to the host immune response as stated in the present study.**

Lines 648 on. This paragraph links the metabolic changes with that of methylation change—however the methylation results are not super convincing. IS this over-reaching?

**We thank the reviewer for this important comment and agree that the link between metabolic and epigenetic changes should be interpreted with caution.**

**“During IIM acquisition, following primary infection and secondary challenge, similar molecular processes were observed in Biomphalaria snails. Hemocytes appear to undergo metabolic reprogramming, likely to meet the energetic demands associated with immune activation, including transcription, differentiation, and proliferation (Fanucchi et al., 2021; Ferreira et al., 2022). In particular, we observed a shift towards aerobic glycolysis, glutaminolysis, and a reduction in oxidative phosphorylation, consistent with a Warburg-like metabolic profile.**

**These metabolic changes, at 24h and 96h after the primary infection, are associated with the accumulation of key metabolites, such as fumarate and other TCA cycle intermediates, which have been shown in vertebrate systems to influence epigenetic enzymes, chromatin modifications and acquisition of trained immunity (Fanucchi et al., 2021, Arts et al., 2016b). In addition, alterations in amino acid metabolism and the urea cycle were observed, pathways that have also been implicated in shaping immune cell function and epigenetic states in vertebrate systems and induce innate immune memory (Wang et al., 2021; Badii et al., 2021, Cabau et al., 2020). Finally, the insulin signaling pathway was also modified in hemocytes, and has been implicated in the regulation of immune cell activation and inflammatory responses (Ieronymaki et al., 2019).**

**While direct evidence linking these metabolic changes to epigenetic reprogramming in Biomphalaria is still lacking, our results suggest that similar mechanisms may be involved. Together, these observations point to a coordinated metabolic and transcriptional reprogramming of hemocytes during IIM acquisition, potentially contributing to enhanced immune response upon secondary challenge.”**

Lines 675-678. This is a strong assertion given the evidence presented.

**We thank the reviewer for this comment. We agree that the original wording was too assertive given the evidence presented. We have therefore revised this section to avoid inferring direct epigenetic modifications or causal mechanisms and instead discuss the observed metabolic changes in the context of pathways previously implicated in immune cell regulation and innate immune memory in other systems.**

Lines 669. The authors claim to have identified central molecular pathways that are indispensable for the induction of innate immune memory. I do not see how they have actually done this. They have found pathways correlated with innate immune memory. Please tone this down.

**We thank the reviewer for this helpful comment and agree that the interpretation of these pathways required a more cautious wording.**

**“Through pathway-level integration of transcriptomic, epigenomic and metabolomic data, we identified central molecular pathways that are consistently associated with the acquisition of innate immune memory in Biomphalaria snail hemocytes. Interestingly, similar pathways have been described in the context of trained immunity in vertebrate innate immune cells.**

**Based on these observations, our results suggest that comparable mechanisms, i.e. “trained immunity”, may operate in *Biomphalaria glabrata* (Gourbal et al., 2018)*.* More broadly, the presence of shared molecular features across different animal lineages supports the hypothesis that trained immunity may represent an evolutionarily conserved trait and hypothesized in previous opinion papers (Penkov et al., 2019, Gourbal et al., 2018).**

**However, our data remain correlative, and further functional studies will be required to determine the extent to which these pathways are directly involved in the induction of innate immune memory in this system.”**

Grammar:

**We thank the reviewer for these helpful comments. Minor grammatical and stylistic corrections have been addressed directly in the manuscript. Detailed responses are provided below for comments requiring clarification:**

There are many grammatical and punctuation errors throughout the paper. I have listed many below. The authors should watch for use of plural v. singular as well as use of tense. There is mixing between tenses in the narrative.

Throughout the paper “infection kinetics” would be better than “kinetic of infection”. When “kinetic” is used as a noun, it is typically used in the plural as “kinetics”. In the paper some alternatives might be considered like time point or time course. **Corrected accordingly**

Also, for secondary challenge, the use of “challenge” is not adequate. Even the primary infection could be considered an infection “challenge”. Thus, it would be less confusing to the reader to specifically state “secondary challenge”.**Corrected accordingly**

Line 67. “evidences” should be “evidence”**Corrected accordingly**

Lines 71-72. “primed in a sustainable manner” is a confusing phrase. I recommend removing “sustainable manner”. Then explain what this phrase was supposed to mean like perhaps: immune memory peaked around 14 days post infection and was sustained for at least 8 -weeks.

**We thank the reviewer for this comment. We agree that the expression “in a sustainable manner” may be confusing in this context. As the sentence is intended to describe the general phenomenon of immune priming across invertebrates rather than the duration of the response in a specific model, we removed this expression from the revised manuscript to improve clarity.**

This is seemingly small, but this change would enhance readability greatly—the use of “P” for primary infection works well, but the use of “C” for the secondary infection does not. “C” is so often linked with “control” and so I had to flip back and forth a lot to reconcile this difference. And I’m finally realizing after many hours of review that C must have been chosen for challenge. However challenge could also be used to describe the primary challenge so not ideal.

**We thank the reviewer for this comment. We acknowledge that the abbreviation “C” may not be immediately intuitive. However, we chose “C” to denote the secondary challenge and have clarified this terminology in both the Materials and Methods and Results sections, where “P” is defined as primary infection and “C” as secondary challenge. To avoid extensive modifications throughout the manuscript and figures, we have now retained this nomenclature while ensuring that it is clearly defined at its first occurrence.**

Line 149 “step” should be “steps”**Corrected accordingly**

Line 127 “increase” should be “increased”**Corrected accordingly**

Line 129, The use of “Only” is confusing—is this the “only” time point where the effect occurs, or are the authors referring to 24 hours as a short amount of time?

**We thank the reviewer for this comment. We have revised the sentence to improve clarity and avoid ambiguity by replacing “Only” with “As early as”.**

Line 132. Were should be was. (or increase to increases) **Corrected accordingly**

Line 141, Awkward construction. Perhaps just “there were no significant differences in counts of the 4 hemocyte types across time points.” **We thank the reviewer for this suggestion. We have revised the sentence accordingly for clarity.**

Line 174. I think it should be “differentially regulated” not just regulated. **Corrected accordingly**

Line 177 “kinetic” should be “time” **Corrected accordingly**

Line 208. Should this read is “still activated” rather than just “activated”. The former suggests genes that are activated during the primary response and remains upregulated during the secondary challenge. The latter would indicated that the sustained response is activated at the two week mark (so not during primary infection)

**We thank the reviewer for this insightful comment. We have revised the sentence to clarify that the immune response remains active two weeks after primary infection.**

**“A sustained immune response remains activated two weeks after primary infection.”**

Line 513 hemolymphe should be hemolymph **Corrected accordingly**

Line 514 “infection s” should be “infections” **Corrected accordingly**

Line 535 “works” should be work **Corrected accordingly**

Line 570 Linked rater than link **Corrected accordingly**

Line 576 “were” should be “Are” and in line 577. **Corrected accordingly**

Line 628 Infection s” to “infections” **Corrected accordingly**

Line 652. “reversal of ATP production” should be “reduction of ATP production”

**We thank the reviewer for this comment. We have revised the sentence to clarify that we refer to a shift in ATP production rather than a reduction.**

**“First, we observed a shift in ATP production in hemocytes towards specific pathways such as aerobic glycolysis, glutaminolysis, and shut down of OxPhos mitochondrial respiratory chain (known as Warburg effect) (Figure 12).”**

Line 672 occurs should be occur **Corrected accordingly**

Line 748. Duplicate, not duplicates. Does this sentence mean that there were duplicate pools or that the snails of one pool were bled twice?

**In this context, ‘duplicate’ refers to a duplicate of two separate mollusc pools**

Line 793. When should be while **Corrected accordingly**

Line 794. Secondary should be secondarily. **Corrected accordingly**

Line 803. Perhaps “Pellets of nuclei were immediately submitted…” **Corrected accordingly**

**In addition to the changes described above, we have made the following modifications to the supplementary materials and figures:**

**Supplementary Tables**

- **Table S1: Added the unit "(mm)" to the "Size" columns in Sheet 1 (*Total_hemo_count*). Sheet 2 was reformatted into a long-table format.**
- **Table S9: Corrected the internal table numbering (previously labeled as Table S8).**
- **Table S10: Corrected the internal table numbering (previously labeled as Table S9).**

**Figures**

- **Former Figures 2 and 3 have been merged into the new Figure 2.**
- **Former Figure 4 and Figure 10 have been redesigned to comply with copyright requirements and is now presented as Figure 3 and Figure 7 respectively.**
- **Former Figures 5 and 6 have been merged into Figure 4.**
- **Former Figures 7 and 8 have been merged into Figure 5.**
- **Former Figure 9 is now Figure 6.**
- **Former Figure 10 is now Figure 7.**
- **Former Figure 11 is now Figure 8.**
- **Former Figure 12 has been removed from the manuscript.**

**In addition, the merged figures were simplified and unnecessary illustrations were removed to improve clarity and readability.**
