# Supplementary material for: Epigenetic and metabolic reprogramming of innate immune cells establishes immunological memory in the Schistosomiasis vector snail Biomphalaria glabrata
Source: PLoS Pathog. 2026 Aug 5;22(8):e1014471. doi: 10.1371/journal.ppat.1014471 (PMC13466059; doi:10.1371/journal.ppat.1014471)
Supplement: Peer review history

# Author comment: Author response to Decision Letter 0

**DOI:** 10.1371/journal.ppat.1014471.r001

**Transfer Alert**

This paper was transferred from another journal. As a result, its full editorial history (including decision letters, peer reviews and author responses) may not be present.

11 Feb 2026

# Peer review: Decision Letter 0

**DOI:** 10.1371/journal.ppat.1014471.r002

**Authors:** Margaret A Phillips, Frédéric Chevalier

23 Mar 2026

PPATHOGENS-D-26-00259

Epigenetic and metabolic reprogramming of innate immune cells establishes immunological memory in Biomphalaria glabrata the vector snail of Schistosomiasis.

PLOS Pathogens

Dear Dr. GOURBAL,

Thank you for submitting your manuscript to PLOS Pathogens. After careful consideration, we feel that it has merit but does not fully meet PLOS Pathogens's publication criteria as it currently stands. Therefore, we invite you to submit a revised version of the manuscript that addresses the points raised during the review process.

Please submit your revised manuscript by May 22 2026 11:59PM. If you will need more time than this to complete your revisions, please reply to this message or contact the journal office at plospathogens@plos.org. When you're ready to submit your revision, log on to https://www.editorialmanager.com/ppathogens/ and select the 'Submissions Needing Revision' folder to locate your manuscript file.

Please include the following items when submitting your revised manuscript:

* A letter that responds to each point raised by the editor and reviewer(s). You should upload this letter as a separate file labeled 'Response to Reviewers'. This file does not need to include responses to any formatting updates and technical items listed in the 'Journal Requirements' section below.

* A marked-up copy of your manuscript that highlights changes made to the original version. You should upload this as a separate file labeled 'Revised Manuscript with Track Changes'.

* An unmarked version of your revised paper without tracked changes. You should upload this as a separate file labeled 'Manuscript'.

If you would like to make changes to your financial disclosure, competing interests statement, or data availability statement, please make these updates within the submission form at the time of resubmission. Guidelines for resubmitting your figure files are available below the reviewer comments at the end of this letter.

We look forward to receiving your revised manuscript.

Kind regards,

Frédéric Chevalier, Ph.D.

Guest Editor

PLOS Pathogens

Margaret Phillips

Section Editor

PLOS Pathogens

Sumita Bhaduri-McIntosh

Editor-in-Chief

PLOS Pathogens

orcid.org/0000-0003-2946-9497

Michael Malim

Editor-in-Chief

PLOS Pathogens

orcid.org/0000-0002-7699-2064

**Additional Editor Comments :**

Expert reviewers have evaluated your manuscript and consider that your study provides some very interesting clues regarding the functional aspects of immune priming in Biomphalaria snails. However, they also identified some weaknesses in the manuscript that need to be addressed.

For example, reviewers suggested that you:

- Edit the introduction to clarify the study hypotheses and better reflect the current state of the field.

- Include additional literature relevant to the field.

- Revise the discussion to include additional interpretations of the results and use a more cautious tone.

- Revise the number of figures to ensure that they are relevant for conveying the main results described in the manuscript.

The comments of the reviewers are enclosed for your consideration. I hope the information provided will be helpful for improving your manuscript.

Sincerely,

Frédéric Chevalier

**Journal Requirements:**

1) Please provide an Author Summary. This should appear in your manuscript between the Abstract (if applicable) and the Introduction, and should be 150-200 words long. The aim should be to make your findings accessible to a wide audience that includes both scientists and non-scientists. Sample summaries can be found on our website under Submission Guidelines:

https://journals.plos.org/plospathogens/s/submission-guidelines#loc-parts-of-a-submission

2) We do not publish any copyright or trademark symbols that usually accompany proprietary names, eg ©,  ®, or TM  (e.g. next to drug or reagent names). Therefore please remove all instances of trademark/copyright symbols throughout the text, including:

- ® on pages: 21, and 22.

3) Please upload all main figures as separate Figure files in .tif or .eps format. For more information about how to convert and format your figure files please see our guidelines:

https://journals.plos.org/plospathogens/s/figures

4) Some material included in your submission may be copyrighted. According to PLOS's copyright policy, authors who use figures or other material (e.g., graphics, clipart, maps) from another author or copyright holder must demonstrate or obtain permission to publish this material under the Creative Commons Attribution 4.0 International (CC BY 4.0) License used by PLOS journals.

Please respond directly to this email and provide any known details concerning your material's license terms and permissions required for reuse, even if you have not yet obtained copyright permissions or are unsure of your material's copyright compatibility. Once you have responded and addressed all other outstanding technical requirements, you may resubmit your manuscript within Editorial Manager.

Potential Copyright Issues:

i) Please confirm (a) that you are the photographer of 1, or (b) provide written permission from the photographer to publish the photo(s) under our CC BY 4.0 license.

ii) Figures 1, 4, and 12. Please confirm whether you drew the images / clip-art within the figure panels by hand. If you did not draw the images, please provide (a) a link to the source of the images or icons and their license / terms of use; or (b) written permission from the copyright holder to publish the images or icons under our CC BY 4.0 license. Alternatively, you may replace the images with open source alternatives. See these open source resources you may use to replace images / clip-art:

- https://commons.wikimedia.org

- https://openclipart.org/.

5) Thank you for stating that "Data are accessible on the repository SRA (sequence read archive) from NCBI under the BioProject ID PRJNA1422672."  Please note that, though access restrictions are acceptable now, your entire data will need to be made freely accessible if your manuscript is accepted for publication. This policy applies to all data except where public deposition would breach compliance with the protocol approved by your research ethics board. If you are unable to adhere to our open data policy, please kindly revise your statement to explain your reasoning and we will seek the editor's input on an exemption. Please be assured that, once you have provided your new statement, the assessment of your exemption will not hold up the peer review process.

6) Please amend your detailed Financial Disclosure statement. This is published with the article. It must therefore be completed in full sentences and contain the exact wording you wish to be published.

1) State what role the funders took in the study. If the funders had no role in your study, please state: "The funders had no role in study design, data collection and analysis, decision to publish, or preparation of the manuscript."

7) Please ensure that the funders and grant numbers match between the Financial Disclosure field and the Funding Information tab in your submission form. Note that the funders must be provided in the same order in both places as well.

Note: If the reviewer comments include a recommendation to cite specific previously published works, please review and evaluate these publications to determine whether they are relevant and should be cited. There is no requirement to cite these works unless the editor has indicated otherwise.

**Reviewers' Comments:**

**Comments to the Authors:**

**Please note that one review is uploaded as an attachment.**

Reviewer's Responses to Questions

**Part I - Summary**

Please use this section to discuss strengths/weaknesses of study, novelty/significance, general execution and scholarship.

Reviewer #1: Memory within innate immune systems is an important phenomenon. It comes as 'trained immunity' in vertebrates and 'immune priming' in invertebrates. The latter consists of a large variety of mechanisms in the different invertebrat taxa. In most cases where phenomena of immune priming have been shown, the underlying mechanisms are not known in details. The present study thus provides an important step towards such better mechanistic knowledge. It specifically asks whether mechanisms that are underlying vertebrate trained immunity might also be present during some phenomena of invertebrates immune priming. The authors make use of a powerful system for studying immune priming, namely the snail Biomphalaria glabrata and its trematode parasite Schistosoma mansoni. Using a combination of hemocyte RNA sequencing, bisulfite sequencing, ATAC sequencing and metabolomics, they indeed find strong indications that metabolic and epigenetic reprogramming of hemocytes seem to occur, pointing towards similarities with vertebrate trained innate immune cells.

Given the almost complete lack of previous evidence for such similarities, these results are novel and highly significant. Another strength of the study is the model system, becasue it consists of a natural host-parasite system. However, this is also bound to a certain (although minor) weakness, because priming is induced by infection, and it is not possible to disentangle infection effects from priming that might occur through non-infectious agents.

The experiments are generally well-executed, with one larger limitation: there doesn't seem to be non-infected controls taken at the same time points as the samples that were taken after priming and challenge. Rather, it seems that all comparisons were done with naive snails from the first time point. At least this is what seems shown in Fig. 1. Such a design would not control for potential changes over time that could have occurred in naive snails as well.

The results are presented and discussed in an appropriate manner, but I do have a minor concerns regarding the introduction and the somewhat limited focus of the discussion that doesn't sufficiently account for alternative explanations.

Overall, this is a novel and highly significant study that is well-suited for the journal PLoS Pathogens.

Reviewer #2: See attachment

Reviewer #3: I will begin my review by pointing out that I do not have personal expertise with many of the methods used in this paper, although I understand them. So, I am taking their experimental results at face value. If there are methodological flaws, I defer to the opinion of other reviewers on that. I can comment on the overall experimental design and the importance of the results.

I thought the experimental design (Fig. 1) was nice in that it allowed them to define when in the process of first and second challenges, over 16 days, the major changes in gene expression and epigenetic modifications take place. Combining RNAseq, ATAC-seq, WGBS and the targeted metabolomics allowed them to take a comprehensive look at the changes involved in the shift to a humoral response seen in IIM. They found substantial epigenetic and metabolomic changes after the first infection, but not the second. After the second challenge there is a massive upregulation in expression of many genes. All the methods point to shifts in cell metabolism, similar to what is observed in vertebrate trained immunity, as key processes in snail IIM.

What type of epigenetic change reprogrammed those cells to respond that way still needs to be explored. As is typical, DNA methylation changes didn’t correlate with gene expression changes. However, the ATAC-seq results were quite interesting. Opening sites overlapped mostly with genes upregulated in their “sustained response” profile, and less so with the “immune shift” response, which I don’t think was expected. There are still many questions to be answered about epigenetic control, but this study was a nice start.

The author speculate quite a bit in the Discussion. I have no problem with that, as I think that is the appropriate place to lay out new ideas and to propose hypotheses to test in the future. However, to avoid criticism, they might want to use more circumspect language, couching anything that isn’t a concrete result with phrases such as “It appears that …”, or “We speculate that…” or “We hypothesize that…”.

This paper appears to be the first on molluscs that investigates how IIM is achieved, and is one in what is still a small literature on IIM in invertebrates. That their results point to metabolic shifts similar to what is seen in vertebrate trained immunity was particularly interesting. What they found should be of interest to researchers studying a variety of organisms. Overall, I enjoyed this thought-provoking paper, and don’t see much to criticize.

One small comment is that there are a lot of figures in the manuscript. Some of the figures come across as just pretty pictures, rather than adding substantially to understanding of the results. For example, they might want to explain what is special about the genomic regions displayed in Fig. 5A and 7A & B. Using the figure legends to tell the reader what the reader should take away from each figure can be useful.

**********

**Part II – Major Issues: Key Experiments Required for Acceptance**

Please use this section to detail the key new experiments or modifications of existing experiments that should be absolutely required to validate study conclusions.

Generally, there should be no more than 3 such required experiments or major modifications for a "Major Revision" recommendation. If more than 3 experiments are necessary to validate the study conclusions, then you are encouraged to recommend "Reject".

Reviewer #1: I don't see any new experiments that would be absolutely required to validate the study conclusions. Of course, we could always ask for more, such as functional validation of some key mechanisms. But this would be too much to ask for, given the complexity of the host studied here.

Reviewer #2: See attachment

Reviewer #3: none

**********

**Part III – Minor Issues: Editorial and Data Presentation Modifications**

Please use this section for editorial suggestions as well as relatively minor modifications of existing data that would enhance clarity.

Reviewer #1: I have a few points that could be addressed with minor modifications of the introduction, the presentation of the experimental design and data, as well as the discussion. All of these points can be addressed with relatively minor modifications of the manuscripts and don't need additional experimentation.

(1) I personally don't like how the diversity of immune priming phenomena is presented as a limitation. Phrasing such diversity as a problem creates unnecessary controversy. Diversity of mechanisms underlying immune priming rather is a biological fact. There are many different mechanisms involved (in the many different taxa), so the call for universality (of model organisms studied) and the illlusion that there might be a uniform mechanistic underpinning is doing harm to the field and hinders the power to gain insight into the many different mechanisms. I agree that some immunologists share the scepticism presented in the introduction (this may be mentioned) but in my view, it is not a suitable introduction (and justification of this study). Why not phrasing the open question to what extent trained immunity - like processes are involved also in invertebrate priming? This provides sufficient novelty and doesn't give the wrong impression that all invertebrate immune priming is the same and based on trained immunity - like processes. I know that the authors are well aware of these aspects, so why not also introducing the topic that way?

(2) Related to this, the introduction plays down the increasing knowledge of mechanistic, molecular underpinnings of immune priming in more and more systems. These mechanisms are often taxon-specific and depend on the type of pathogens, route of infection, etc. Why not doing justice to these studies and naming at least some examples? This doesn't decrease the relevance of the question addressed here, namely whether mechanisms similar to trained immunity might also be relevant for invertebrates, which remains to be an exciting an novel question.

(3) The potential limitation in study design mentioned above (no control samples taken at the exact same time point after priming and challenge) should be addressed more clearly. First, it has to become clear from the results and methods description whether this was indeed the case? If so, it should be discussed why this is not regarded a limitation (I assume the authors have arguments, otherwise they would have taken these time-matched controls). Related to this, it doesn't become clear whether any kind of 'sham infection' was done as control?

(4) Please provide more details regarding the infection success. Where all exposed snails infected (when primary infected)? From line 708 it seems not? If that is the case, what happened to exposed-but not infected snails? Where they not studied any further? If not, what are the arguments why not? If many snails were exposed-but not infected, this could bias the subgroup of infected snails and effects might be partially due to this selection effect. I assume the authors have thought about this and have good arguments why this doesn't pose any problems.

(5) It should be acknowledged that proof for epigenetic reprogramming is currently still a bit limited in the system studied here. The role of CpG methylation is unclear (methylation rates are low and unrelated to gene expression). ATAC seq provides valuable information about chromatin accessibility but it doesn't provide the mechanisms causing these differences, such as histone modifications. I am not asking for futher experiments looking into this, because it would be challenging in this system. But these limitations should be spelled out even more clearly. In Fig. 12 epigen. modification are shown that were not studied here (I see that this is described as 'theoretical' but still, why not sticking to what was studied here?)

(6) The discussion is very much focused on trying to proof epigenetic and metabolic reprogramming, which is fair enough, but I would prefer a slightly more open approach. For example, how is the potential involvement of these processes interpreted in the light of previous findings in this system (FREPs etc.?). What about the (lack of) specificity, e.g. for schistosome strains?

(7) The absence of changes in hemocyte types depends on the reliabilty to discriminate hemocytes. It is quite likely, that improved techniques (such as scRNAseq) would differentiate hemocytes that are morphologically indistinguishable (as has been shown in other invertebrates). This is important, because the techniques chosen here describe bulk changes (in the mixed population of hemocytes). It thus remains unstudied here whether observerd changes are due to changes (of expression, of open chromatin, etc) occurring to individual cells, or whether cells with certain expression patterns might become more numerous after priming (while expression of individual cells itself might remain unchanged). This distinction is relevant regarding the interpretation of the results (such as similiarity with trained immunity) and should at least be discussed in more detail.

(8) ATAC sec compared P24h and C24h vs naive while methylation compares P16d vs naive. Why were different timepoints chosen? I guess there are good reasons, please mention them.

There are some further minor points:

Title: either put a comma after 'glabrata' or write '…in the schistosomiasis vector snail Biomphalaria glabrata'.

L. 128: reduce number of digits

L. 146 ff and L. 192 ff. Fig 1 and 3: improve this figure legends, partially awkward style

L. 166: Fig 2B: same N as in Fig2A?

l. 276 and above: rationale for sorting genes into recalled, sustained and shift has to be presented in a clearer manner

l. 290 what does 'mosaic type' mean - please explain

l. 318, interesting point, provide more details (maybe in the discussion): what is known about the function of methylation in snails?

l. 368 the direction of the described effects is unclear; I assume 'opening' means open in treatments (here C24h) compared to control (as it is written, it seems the other direction was meant?)

- l 386 which phenotype? transcriptome?

- l 472: color scheme in fig 10D?

- l. 682 specify in more detail what kind of 'further work'

- Table 1 is unclear and confusing regarding the exact levels of replication, etc.

Some Figures are truncated in pdf but ok in pptx

Fig 3E how were enriched function ‘selected’?

Discussion/Literature:

There is a recent study that also indicates the involvement of epigenetic reprogramming that the authors may wish to cite:

Deng Z, Luo J, Zhang Y, Li C, Deng S, He J, He Z, Wu W, Jiao R, Liu J. 2025. PGRP–SC–mediated innate immune memory against pathogenic bacteria in Drosophila. hLife 1:3. DOI: https://doi.org/10.1016/j.hlife.2025.09.003

Reviewer #2: Lots of grammatical issues. See attachment.

Reviewer #3: see main comments

**********

PLOS authors have the option to publish the peer review history of their article (what does this mean?). If published, this will include your full peer review and any attached files.

If you choose “no”, your identity will remain anonymous but your review may still be made public.

**Do you want your identity to be public for this peer review?**  For information about this choice, including consent withdrawal, please see our Privacy Policy.

Reviewer #1: **Yes:**  Joachim Kurtz

Reviewer #2: No

Reviewer #3: No

[NOTE: If reviewer comments were submitted as an attachment file, they will be attached to this email and accessible via the submission site. Please log into your account, locate the manuscript record, and check for the action link "View Attachments". If this link does not appear, there are no attachment files.]

**Figure resubmission:**

While revising your submission, we strongly recommend that you use PLOS’s NAAS tool (https://ngplosjournals.pagemajik.ai/artanalysis) to test your figure files. NAAS can convert your figure files to the TIFF file type and meet basic requirements (such as print size, resolution), or provide you with a report on issues that do not meet our requirements and that NAAS cannot fix.

After uploading your figures to PLOS’s NAAS tool - https://ngplosjournals.pagemajik.ai/artanalysis, NAAS will process the files provided and display the results in the "Uploaded Files" section of the page as the processing is complete. If the uploaded figures meet our requirements (or NAAS is able to fix the files to meet our requirements), the figure will be marked as "fixed" above. If NAAS is unable to fix the files, a red "failed" label will appear above. When NAAS has confirmed that the figure files meet our requirements, please download the file via the download option, and include these NAAS processed figure files when submitting your revised manuscript.

**Reproducibility:**

To enhance the reproducibility of your results, we recommend that authors of applicable studies deposit laboratory protocols in protocols.io, where a protocol can be assigned its own identifier (DOI) such that it can be cited independently in the future. Additionally, PLOS ONE offers an option to publish peer-reviewed clinical study protocols. Read more information on sharing protocols at https://plos.org/protocols?utm_medium=editorial-email&utm_source=authorletters&utm_campaign=protocols

Attachment Submitted filename: Review of MS.docx

# Author comment: Author response to Decision Letter 1

**DOI:** 10.1371/journal.ppat.1014471.r003

16 Jun 2026

Attachment Submitted filename: Response to reviewers.docx

# Peer review: Decision Letter 1

**DOI:** 10.1371/journal.ppat.1014471.r004

**Authors:** Margaret A Phillips, Frédéric Chevalier

14 Jul 2026

Dear Pr. GOURBAL,

We are pleased to inform you that your manuscript 'Epigenetic and metabolic reprogramming of innate immune cells establishes immunological memory in the Schistosomiasis vector snail Biomphalaria glabrata.' has been provisionally accepted for publication in PLOS Pathogens.

Before your manuscript can be formally accepted you will need to complete some formatting changes, which you will receive in a follow up email. A member of our team will be in touch with a set of requests.

Please note that your manuscript will not be scheduled for publication until you have made the required changes, so a swift response is appreciated.

IMPORTANT: The editorial review process is now complete. PLOS will only permit corrections to spelling, formatting or significant scientific errors from this point onwards. Requests for major changes, or any which affect the scientific understanding of your work, will cause delays to the publication date of your manuscript.

Should you, your institution's press office or the journal office choose to press release your paper, you will automatically be opted out of early publication. We ask that you notify us now if you or your institution is planning to press release the article. All press must be co-ordinated with PLOS.

Thank you again for supporting Open Access publishing; we are looking forward to publishing your work in PLOS Pathogens.

Best regards,

Frédéric Chevalier, Ph.D.

Guest Editor

PLOS Pathogens

Margaret Phillips

Section Editor

PLOS Pathogens

Sumita Bhaduri-McIntosh

Editor-in-Chief

PLOS Pathogens

orcid.org/0000-0003-2946-9497

Michael Malim

Editor-in-Chief

PLOS Pathogens

orcid.org/0000-0002-7699-2064

***********************************************************

Dear Dr. Gourbal,

Thank you for resubmitting your manuscript to PLOS Pathogens (PPATHOGENS-D-26-00259 - "Epigenetic and metabolic reprogramming of innate immune cells establishes immunological memory in Biomphalaria glabrata the vector snail of Schistosomiasis").

Two of the three initial reviewers re-evaluated your manuscript, and I personally evaluated the response to reviewer 2's comments, as that reviewer was unavailable to review this resubmission. All reviewers considered that the comments were addressed adequately. Only minor issues related to spelling and grammar need to be addressed.

In addition, it seems that one of reviewer 2's comments was addressed in the rebuttal letter, but the corresponding change was not made in the revised manuscript. This concerns l. 658: "Epigenetic and metabolic changes coexist in vertebrates" should be "Epigenetic and metabolic changes are tightly linked in vertebrates" to fully address the comment originally made by reviewer 2 for l. 580.

The reviewers' comments are enclosed for your consideration. I hope the information provided will be helpful for improving your manuscript.

Sincerely,

Frédéric Chevalier

Guest Editor

Reviewer Comments (if any, and for reference):

Reviewer's Responses to Questions

**Part I - Summary**

Please use this section to discuss strengths/weaknesses of study, novelty/significance, general execution and scholarship.

Reviewer #1: The authors have improved the manuscript considerably, based on my own and the other reviewers' comments.

Reviewer #3: I think the authors did an acceptable job addressing reviewer concerns, and I have no further comments.

**********

**Part II – Major Issues: Key Experiments Required for Acceptance**

Please use this section to detail the key new experiments or modifications of existing experiments that should be absolutely required to validate study conclusions.

Generally, there should be no more than 3 such required experiments or major modifications for a "Major Revision" recommendation. If more than 3 experiments are necessary to validate the study conclusions, then you are encouraged to recommend "Reject".

Reviewer #1: There are no major issues

Reviewer #3: (No Response)

**********

**Part III – Minor Issues: Editorial and Data Presentation Modifications**

Please use this section for editorial suggestions as well as relatively minor modifications of existing data that would enhance clarity.

Reviewer #1: On a minor note, some further spell and grammar checks are recommended throughout the whole manuscript, please find examples below.

Everywhere, please check the correct usage of future, present, past tense, e.g. l. 77 ‘will be’ is probably not the suitable tense here.

L .74: freshwater snail

l. 77: intense research has…

l. 111: mechanistic underpinnings

l. 113: live

l. 118 have

l. 119 phenomena

l. 146 a highly specific IIM response has been demonstrated…

l. 148 specific, a whole …

l. 162 we hypothesise

Reviewer #3: (No Response)

**********

PLOS authors have the option to publish the peer review history of their article (what does this mean?). If published, this will include your full peer review and any attached files.

If you choose “no”, your identity will remain anonymous but your review may still be made public.

**Do you want your identity to be public for this peer review?**  For information about this choice, including consent withdrawal, please see our Privacy Policy.

Reviewer #1: **Yes:**  Joachim Kurtz

Reviewer #3: No

# Editor report: Acceptance letter

**DOI:** 10.1371/journal.ppat.1014471.r005

**Authors:** Margaret A Phillips, Frédéric Chevalier

Dear Pr. GOURBAL,

We are delighted to inform you that your manuscript, "Epigenetic and metabolic reprogramming of innate immune cells establishes immunological memory in the Schistosomiasis vector snail Biomphalaria glabrata.," has been formally accepted for publication in PLOS Pathogens.

We have now passed your article onto the PLOS Production Department who will complete the rest of the pre-publication process. All authors will receive a confirmation email upon publication.

The corresponding author will soon be receiving a typeset proof for review, to ensure errors have not been introduced during production. Please review the PDF proof of your manuscript carefully, as this is the last chance to correct any scientific or type-setting errors. Please note that major changes, or those which affect the scientific understanding of the work, will likely cause delays to the publication date of your manuscript. Note: Proofs for Front Matter articles (Pearls, Reviews, Opinions, etc...) are generated on a different schedule and may not be made available as quickly.

Soon after your final files are uploaded, the early version of your manuscript, if you opted to have an early version of your article, will be published online. The date of the early version will be your article's publication date. The final article will be published to the same URL, and all versions of the paper will be accessible to readers.

For Research Articles, you will receive an invoice from PLOS for your publication fee after your manuscript has reached the completed accept phase. If you receive an email requesting payment before acceptance or for any other service, this may be a phishing scheme. Learn how to identify phishing emails and protect your accounts at https://explore.plos.org/phishing.

Thank you again for supporting open-access publishing; we are looking forward to publishing your work in PLOS Pathogens.

Best regards,

Sumita Bhaduri-McIntosh

Editor-in-Chief

PLOS Pathogens

orcid.org/0000-0003-2946-9497

Michael Malim

Editor-in-Chief

PLOS Pathogens

orcid.org/0000-0002-7699-2064
